# An axon-intrinsic loop restricts nerve regeneration through axonal protein synthesis

**DOI:** 10.1126/sciadv.aed0049

**Published:** 2026-08-26

**Authors:** Courtney N. Buchanan, Jinyoung Lee, Samaneh Matoo, Jordan A. Headen, Marie-Claire Honoree, Molly Conway, Lillian F. Thompson, Terika P. Smith, Ansley McKay, Cailyn Tosadori, Irene Dalla Costa, Moira Lopez De Leon, Elizabeth Thames, Nora Perrone-Bizzozero, Ashley L. Kalinski, Kristy Welshhans, Lauren S. Vaughn, Jeffery L. Twiss

**Affiliations:** ^1^ Department of Biological Sciences, University of South Carolina; Columbia, SC 29204 USA.; ^2^ Department of Chemistry & Biochemistry, University of Virginia; Charlottesville, VA 22904 USA.; ^3^ Department of Neurosciences, University of New Mexico; Albuquerque, NM 87131 USA.; ^4^ SmartState Center for Childhood Neurotherapeutics; University of South Carolina, Columbia, SC 29204 USA.; ^5^ Carolina Autism and Neurodevelopment Center, University of South Carolina; Columbia, SC 29204 USA.

## Abstract

Injured axons synthesize the RNA Binding Protein KHSRP that promotes mRNA decay and slows nerve regeneration. Axotomy-induced increase in axoplasmic Ca^2+^ activates axonal *Khsrp* translation, and while axonal Ca^2+^ returns to pre-injury levels within 16 hours post-axotomy, axonal KHSRP remains elevated. Alternating translation of *Reg3a* and *Khsrp* sustains KHSRP levels in regenerating axons. Axonal *Reg3a* mRNA and protein increase proximal to the injury site days after sciatic nerve crush. REG3A stimulates ER Ca^2+^ release in axons to activate PERK, increase eIF2α phosphorylation, and increase *Khsrp* translation. Axoplasmic Ca^2+^ slowly oscillates in growth cones of cultured neurons and *Reg3a* depletion attenuates growth cone Ca^2+^ oscillations, decreases KHSRP synthesis and reduces axonal retractive events in cultured neurons, and accelerates peripheral nerve regeneration in vivo. Thus, REG3A regulation of axonal KHSRP synthesis provides a signaling loop that decelerates axon growth through localized mRNA translation.

## INTRODUCTION

Though injured axons in the peripheral nervous system (PNS) can spontaneously regenerate, the rate of axon growth is quite slow at 1–3 mm per day (*1*, *2*). Regeneration over distances of 5 cm or greater is often unsuccessful since the environment within the distal severed nerve loses the ability to support regeneration and target tissues are less receptive for reinnervation (*3*). Accelerating rates of regeneration would allow axons to reach nerve segments well beyond the injury site and innervate target tissues before loss of growth support and tissue receptiveness for reinnervation occur. We recently showed that deletion of the murine KH-type splicing regulatory factor (KHSRP) accelerates nerve regeneration (*4*). KHSRP is a multifunctional RNA binding protein that contributes to RNA splicing, mRNA transport, and mRNA decay (*5*). KHSRP slows axon growth by promoting decay of axonal mRNAs with AU-rich elements (ARE) in their 3′ untranslated regions (UTR), including growth associated *Gap43*, *Snap25*, and *Prenly-Cdc42* mRNAs (*4*, *6*). *Khsrp* mRNA translation is acutely activated in proximal axons after nerve injury through a Ca^2+^ → PERK→eIF2α^PS51^ signaling cascade (*4*). However, axonal translation switches from mRNAs that are efficiently translated with elevated axoplasmic Ca^2+^, like *Khsrp* and *Kpnb1*, to mRNAs that are more efficiently translated at lower axoplasmic Ca^2+^, like *Csnk2a1* and *Nrn1*, over 16 h after axotomy (*4*, *7*). Despite that switch in translation, axonal KHSRP levels remain elevated in peripheral nerve axons for weeks after axotomy. How are axonal KHSRP levels sustained if *Khsrp* mRNA translation requires elevated axoplasmic Ca^2+^?

We previously showed that *Regenerating Family Member 3*α (*Reg3a*) mRNA levels increase in regenerating axons (*8*) and initial experiments here pointed to an axon growth-attenuating role for REG3A protein similar to KHSRP. REG3A [also called Pancreatitis-associated protein 2 (PAP2) and Lithostathine 3] is a secreted lectin-like protein that was first implicated in pancreatic islet cell regeneration where its expression is increased (*9*). REG3A has been suggested to signal through cell surface EXTL3 receptors but it also has reported bactericidal activity by generating membrane pores (*10*–*13*). Here, we show that local synthesis of REG3A in injured nerves activates translation of axonal *Khsrp* mRNA in regenerating axons to slow PNS nerve regeneration. *Reg3a* depletion accelerates regeneration of both sensory and motor axons in vivo pointing to a previously unrecognized mechanism that impairs nerve regeneration.

## RESULTS

### REG3A protein slows axon growth

**Fig. 1.**
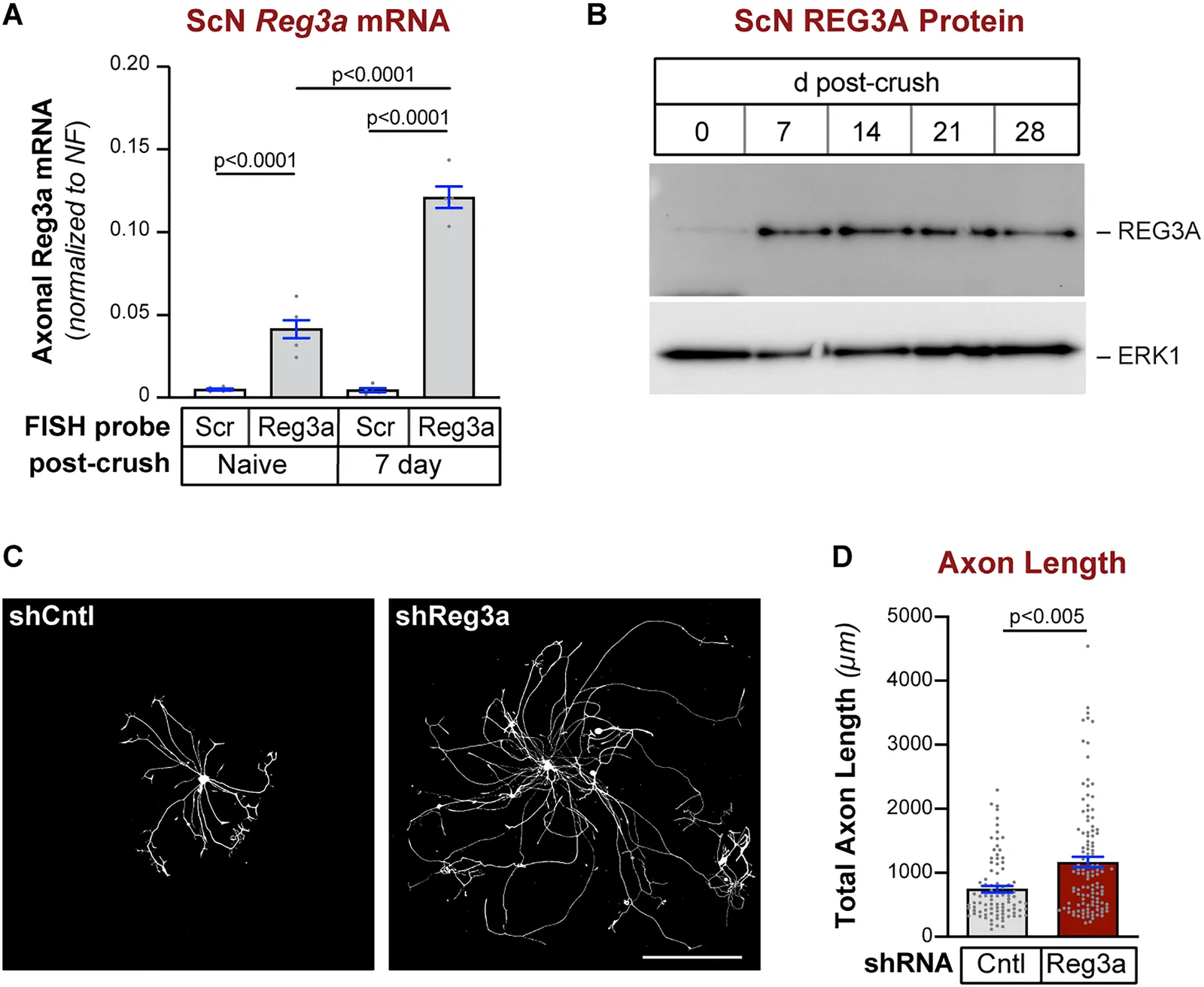
Axon injury increases axonal REG3A mRNA and protein. (**A**) Quantitation of smFISH *Reg3a* signals for naïve vs. 7 d crush injured sciatic nerve (ScN). One-way ANOVA with Tukey post-hoc analysis, mean ± SEM (*n* ≥ 4 mice per group). (**B**) Representative immunoblot of 0–28 d post-injury ScN lysates shows increase in REG3A with relatively equivalent levels of ERK1 protein as loading control. (**C** and **D**) Images for SMI312 (NF) + TuJ1 in AAV-shCntl vs. -shReg3a transduced adult DRG cultures (C). Quantitation shows increased axon length with shReg3a vs. shCntl (D). Student’s *t* test, mean ± SEM (*n* ≥ 75 neurons across ≥3 replicate cultures); scale bar, 100 μm.

Axonal *Reg3A* mRNA levels increase in regenerating peripheral nerve and ascending spinal cord axons after axotomy (*8*). Consistent with that previous report, we find that *Reg3a* mRNA increases in proximal sciatic nerve axons at 7 d following crush injury (Fig. 1A). Immunoblots of lysates of injured sciatic nerve from proximal to the injury site further show increased REG3A protein over 7–28 d after nerve crush injury (Fig. 1B). We used shRNA depletion to determine if the axonal *Reg3A* increase might contribute to neurite growth. shRNA specificity and efficiency were evaluated in adult mouse dorsal root ganglia (DRG) cultures transduced with adeno-associated virus, serotype 9 (AAV)-shRNA targeting *Reg3a* (shReg3a). shReg3a transduced DRG cultures showed decrease in both *Reg3a* mRNA and REG3A protein compared to those transduced with non-targeting shRNA (shCntl) transduction (fig. S1, A and B). Neurite lengths and branching were increased in the shReg3a-compared to those shCntl-transduced DRG cultures (Fig. 1, C and D, and fig. S1C). Note that the neurites extended by cultured DRG neurons show morphological features of axons, including microtubule polarity, axon-restricted kinesin isoforms and absence of dendrite markers (*14*–*17*)—thus, we refer to the DRG neurites as axons herein. Thus, the findings above raise the possibility that the elevation of *Reg3a* mRNA after PNS injury might attenuate axon growth.

**Fig. 2.**
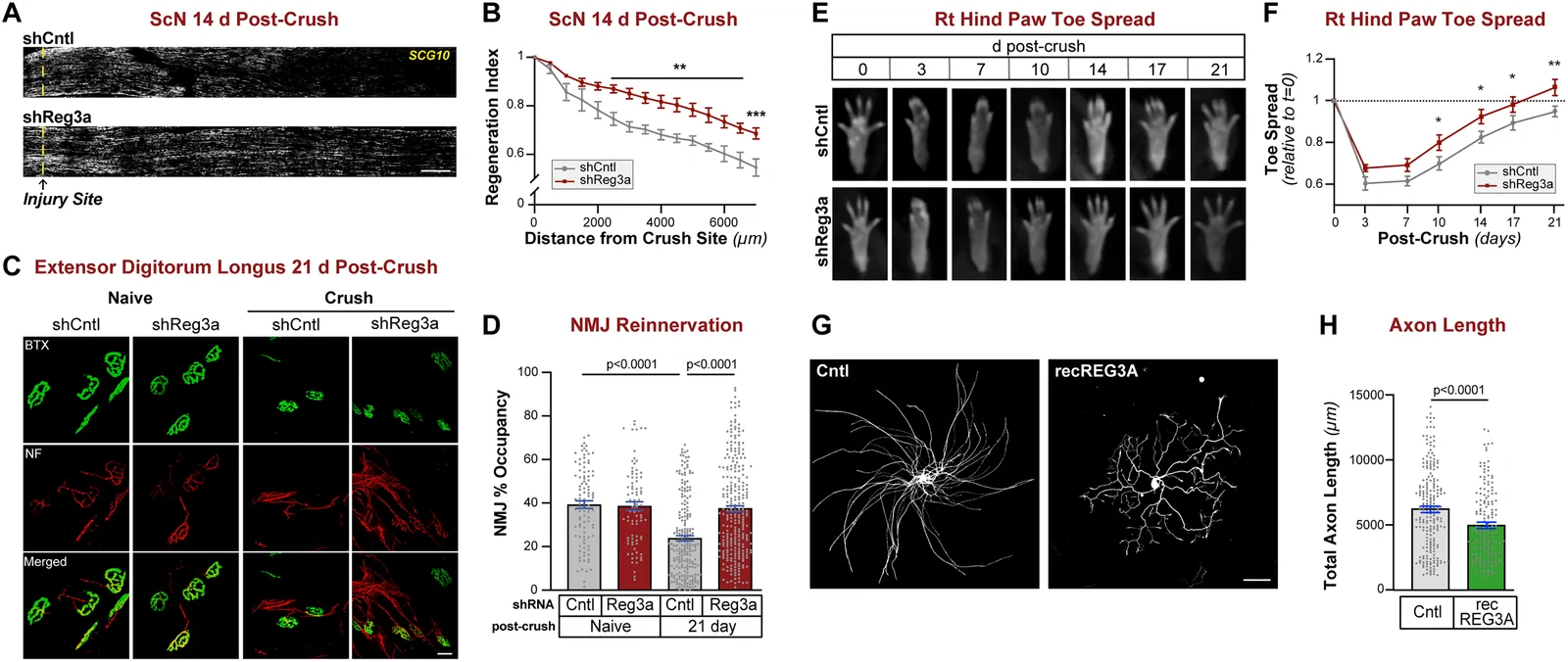
REG3A slows axon growth and regeneration. (**A** and **B**) Exposure matched SCG10 images from ScN at 14 d post-nerve crush from AAV-shReg3a vs. -shCnt transduced mice [(A); left is proximal and right is distal]. (B) Regeneration indices for mice as in (E). Two-way ANOVA with Sidak post-hoc analysis, mean ± SEM, **P ≤ 0.01 and ***P ≤ 0.005 (*n* ≥ 5 per group); scale bar, 500 μm. (**C** and **D**) Images for NMJs in extensor digitorum longus (EDL) of shCntl vs. shReg3a transduced mice at 0 and 21 d post ScN crush injury (C). Quantitation of NMJ occupancy shown in (D). Two-way ANOVA with Tukey post-hoc analysis (*n* ≥ 84 NMJs across 6 animals; mean ± SEM; scale bar, 20 μm. (**E** and **F**) Representative images of right (Rt) hind paw (E) from behavioral analyses of AAV-shRNA transduced mice shReg3a expressing mice show increased toe spreading beginning. Quantitation of toe spread shown in (F). Two-way ANOVA with Tukey post-hoc analysis, mean ± SEM, *P ≤ 0.05 and **P ≤ 0.005 (*n* = 10 shCntl and 13 shReg3A). (**G** and **H**) SMI312 + TuJ1 images for control vs. recREG3A treated adult DRG cultures (G). Quantitation of axon morphologies shows decreased length and increased branching after recREG3A exposure (H). Student’s *t* test, mean ± SEM (*n* ≥ 163 neurons); scale bar, 100 μm.

We used in vivo *Reg3a* depletion to determine if REG3A protein impairs PNS axon regeneration. For this, mice were transduced with AAV9-shReg3a-BFP or -shCntl-BFP by direct injection into the sciatic nerve. Sciatic nerves of shReg3a-transduced mice showed clear in vivo reduction of intra-axonal *Reg3a* mRNA, showing the AAV-shReg3a specifically depletes neuronal *Reg3a* (fig. S1, D to F). Intra-axonal REG3A immunoreactivity was also reduced in the shReg3a-transduced mice (fig. S1, G and H). Over 7–28 d post nerve crush, the shCntl-transduced mice showed increased *Reg3a* mRNA in the L3–5 DRGs and sciatic nerve following crush injury by RTddPCR (fig. S1, I and J), as we previously reported for non-transduced mice (*8*). Sciatic nerves and L3–5 DRGs of the shReg3a-transduced mice showed decreased *Reg3a* mRNA at each time point post-injury compared to shCntl-transduced mice (fig. S1, I and J). Nerve regeneration indices increased in the Reg3a-depleted mice, with greatest difference compared to shCntl-transduced mice at 14 d post-injury (Fig. 2, A and B, and fig. S1, K to M). The shReg3a-transduced mice also showed increased neuromuscular junction occupancy at 21 d post-nerve injury, suggesting accelerated reinnervation of target tissues following nerve injury (Fig. 2, C and D).

We next asked whether *Reg3a* depletion affects functional reinnervation of synaptic targets after sciatic nerve injury using the BlackBox Bio system. This instrument measures behaviors of freely moving mice in a dark environment using continuous recording of paw placement and pressure as a measure for limb usage (*18*). To initially test ability to assess nerve regeneration using this approach, we analyzed KHSRP knockout mice, since *KHSRP* deletion accelerates PNS regeneration compared to wild type mice (*KHSRP^−/−^* and *KHSRP^+/+^*, respectively) (*4*). Consistent with previous findings (*19*), *KHSRP^−/−^* mice showed increased motility based on distance traveled before injury compared to wild type mice (fig. S2A). After right-sided sciatic nerve crush, the *KHSRP^−/−^* mice further showed accelerated functional recovery based on right to left hind paw luminance and right to average front paws luminance compared to *KHSRP^+/+^* mice (fig. S2, B to D). Since paw luminance showed the most consistent differences at 21 d and earlier after injury in *KHSRP^−/−^* vs. *KHSRP^+/+^* mice, we focused on 0–21 d post-crush interval for assessing recovery in REG3A*-*depleted mice. For this, sciatic nerves of wild type mice were bilaterally injected with AAV-shReg3a-BFP or -shCntl-BFP (for transduction of motor and sensory neurons) 14 d prior to a right-sided sciatic nerve crush. The AAV-shCntl- and -shReg3a-transduced mice showed no differences in motility (fig. S2E). Right hind paw toe spreading, right to left hind paw luminance, and right hind to average front paw luminance were increased in the shReg3a- vs. shCntl-transduced over 10–21 d post-injury (Fig. 2, E and F, and fig. S2, F to H; note – *KHSRP^−/−^* mice chewed their ipsilateral hind paw toes after crush injury, so toe spreading could not be assessed in the constitutive KHSRP knockout). Thus, based on both morphological and functional assessments, REG3A depletion accelerates peripheral nerve regeneration.

### REG3A reduces axon regeneration through translational modulation of axonal Khsrp mRNA in cultured neurons

**Fig. 3.**
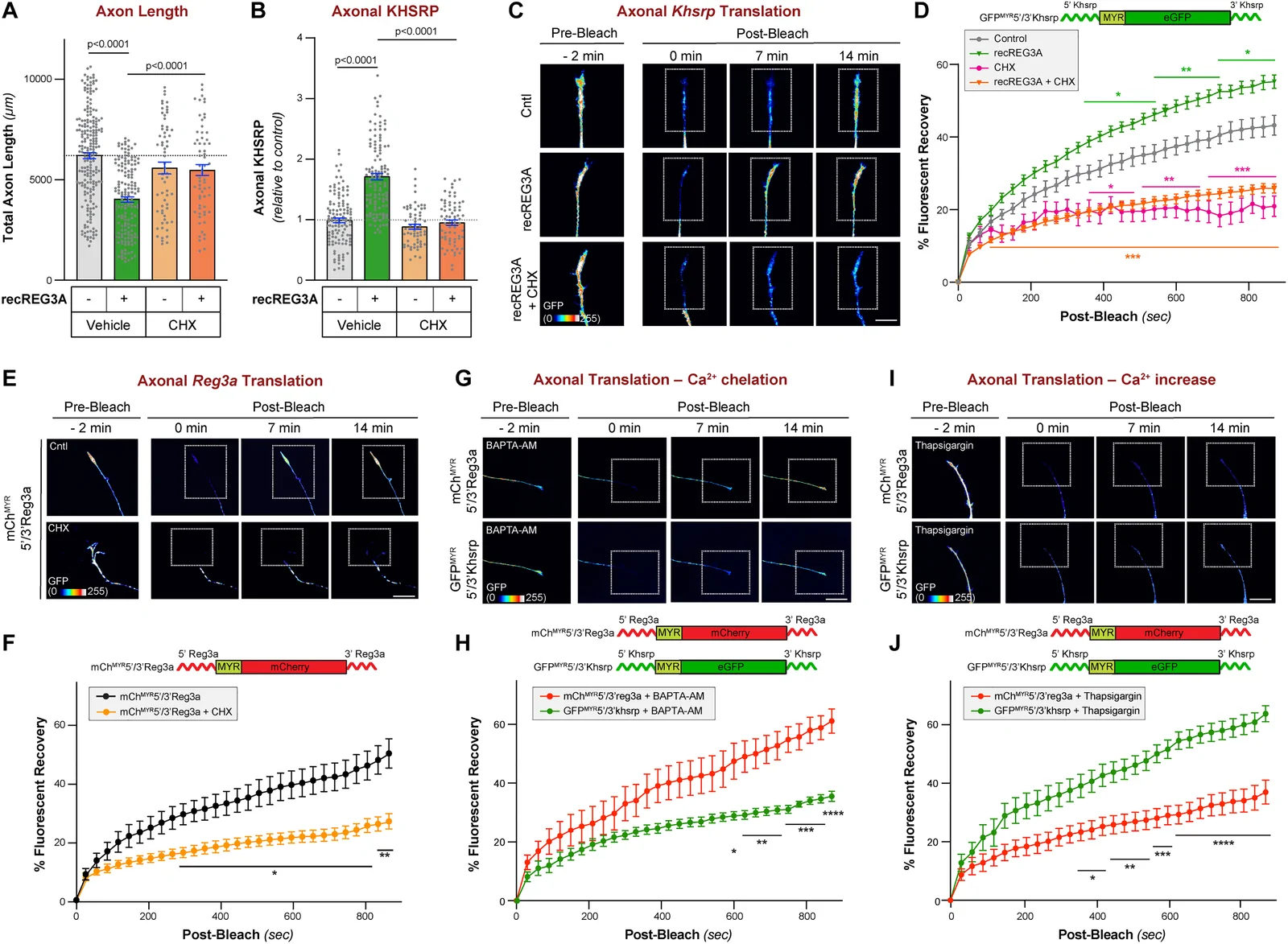
REG3A increases axonal translation of Khsrp mRNA. (**A** and **B**) Axon length (A) and axonal KHSRP (B) for DRG neurons treated ± recREG3A and vehicle (DMSO) vs. cycloheximide (CHX). One-way ANOVA with Tukey post-hoc analysis, mean ± SEM (*n* ≥ 63); scale bar, 100 μm. (**C** and **D**) FRAP terminal axon GFP^MYR^5’/3’Khsrp in transfected DRGs treated with recREG3A ± CHX shown as representative image sequences (C) and quantitation (D). Two-way ANOVA with Tukey post-hoc analysis, mean ± SEM, *P ≤ 0.05, **P ≤ 0.005, and ***P ≤ 0.0005, with colors matched to dataset vs. control (*n* ≥ 8 axons across ≥3 culture preparations); scale bar, 10 μm. (**E** and **F**) Image sequences (E) and quantitation (F) for axonal FRAP in mCh^MYR^5’/3’Reg3a transfected DRGs ± CHX. Repeated measures ANOVA with Tukey post-hoc analysis, *P ≤ 0.05 and **P ≤ 0.01, mean ± SEM (*n *≥ 10 axons across ≥3 culture preparations); scale bar, 10 μm. (**G** to **J**) Image sequences (G and I) and quantification (H and J) for axonal FRAP in mCh^MYR^5’/3’Reg3a + GFP^MYR^5’/3’Khsrp transfected DRGs treated with BAPTA-AM to chelate intracellular Ca^2+^ (G and H) or Thapsigargin to increase intracellular Ca^2+^ (I and J). Mean ± SEM, repeated measures ANOVA with Tukey post-hoc analysis, *P ≤ 0.05, **P ≤ 0.01, ***P ≤ 0.005, and ****P ≤ 0.00; scale bar, 10 μm.

The accelerated nerve regeneration seen with REG3A depletion mirrors what we recently published for *KHSRP* knockout mice (*4*). Since *Reg3a* mRNA encodes a lectin-like protein that includes an N-terminal signal peptide for secretion (GenBank ID # NM_011259), we asked if recombinant REG3A (recREG3A) protein affects axon growth. In contrast to *Reg3a*-depletion, bath applied recREG3A reduced axon length and increased axon branching in adult DRG cultures (Fig. 2, G and H, and fig. S1N). These changes in axon growth triggered by recREG3A were prevented by pretreatment with the protein synthesis inhibitor cycloheximide (CHX; Fig. 3A and fig. S3, A and B). RecREG3A also increased axonal KHSRP levels and this was prevented by CHX pretreatment (Fig. 3B and fig. S3C).

We previously used fluorescence recovery after photobleaching (FRAP) of diffusion-limited GFP reporter mRNA (GFP^MYR^) that includes the 5′ and 3’ UTRs of *Khsrp* mRNA (GFP^MYR^5’/3’Khsrp) as a surrogate to test for axonal translation of *Khsrp* (*4*). Thus, we asked if axonal translation of GFP^MYR^5’/3’Khsrp is affected by recREG3A. By FRAP analyses, translation of *GFP^MYR^5’/3’Khsrp* was elevated in distal axons in response to recREG3A compared to untreated cultures, and this was attenuated by CHX treatment (Fig. 3, C and D). This suggests that the increase in KHSRP seen in response to recREG3A is through intra-axonal translation of *Khsrp* mRNA.

With recREG3A decreasing axon growth and increasing axonal KHSRP, we asked if recREG3A ‘s effects on axon growth require KHSRP. The axon growth attenuating effects of recREG3A were not seen in DRGs cultured from *KHSRP^−/−^* mice (fig. S3D), indicating KHSRP is required for recREG3A’s axon growth attenuating effect. Axonal *Calreticulin* mRNA (*Calr*) is translated in response to elevated axoplasmic Ca^2+^ similar to axonal *Khsrp* (*4*, *20*, *21*). recREG3A treatment still increased axonal CALR protein levels in the *KHSRP^−/−^* DRG cultures (fig. S3E). Together, these data suggest that REG3A decreases axon growth by increasing axonal translation of *Khsrp* mRNA, and they raise the possibility that this occurs through Ca^2+^ signaling.

### Axonal Reg3a and Khsrp mRNAs show opposite translational regulation by Ca^2+^ in cultured neurons

Consistent with endogenous *Reg3a* mRNA’s localization into axons, a myristoylated mCherry (mCh^MYR^) fluorescent reporter with *Reg3a*’s 5′ and 3’UTRs (mCh^MYR^5’/3’Reg3a) is locally translated in axons of cultured DRG neurons by FRAP analyses (Fig. 3, E and F). Since *Khsrp* mRNA translation is increased by elevated axoplasmic Ca^2+^ and decreased by intracellular Ca^2+^ chelation (*4*), we asked if axonal *mCh^MYR^5’/3’Reg3a* translation is similarly sensitive to axoplasmic Ca^2+^ modulation using FRAP for the *Reg3a* translation reporter. In DRG cultures co-expressing *mCh^MYR^5’/3’Reg3a* and *GFP^MYR^5’/3’Khsrp*, axonal mCh^MYR^5’/3’Reg3a fluorescent recovery was significantly higher upon exposure to the intracellular Ca^2+^-chelator BAPTA-AM compared to GFP^MYR^5’/3’Khsrp (Fig. 3, G and H). In contrast, axonal mCh^MYR^5’/3’Reg3a fluorescent recovery was significantly decreased by thapsigargin, which increases cytoplasmic Ca^2+^ levels by blocking Ca^2+^ uptake by the ER, compared to GFP^MYR^5’/3’Khsrp’s (Fig. 3, I and J). The elevation of GFP^MYR^5’/3’Khsrp translation with thapsigargin and depressed translation of GFP^MYR^5’/3’Khsrp with intracellular Ca^2+^ chelation is consistent with previous findings (*4*). Together, these data argue that *Reg3a* and *Khsrp* mRNAs are translated under distinctly different intracellular Ca^2+^ concentrations.

### REG3A stimulates release of axonal Ca^2+^ stores and activates an intrinsic stress response in cultured neurons

**Fig. 4.**
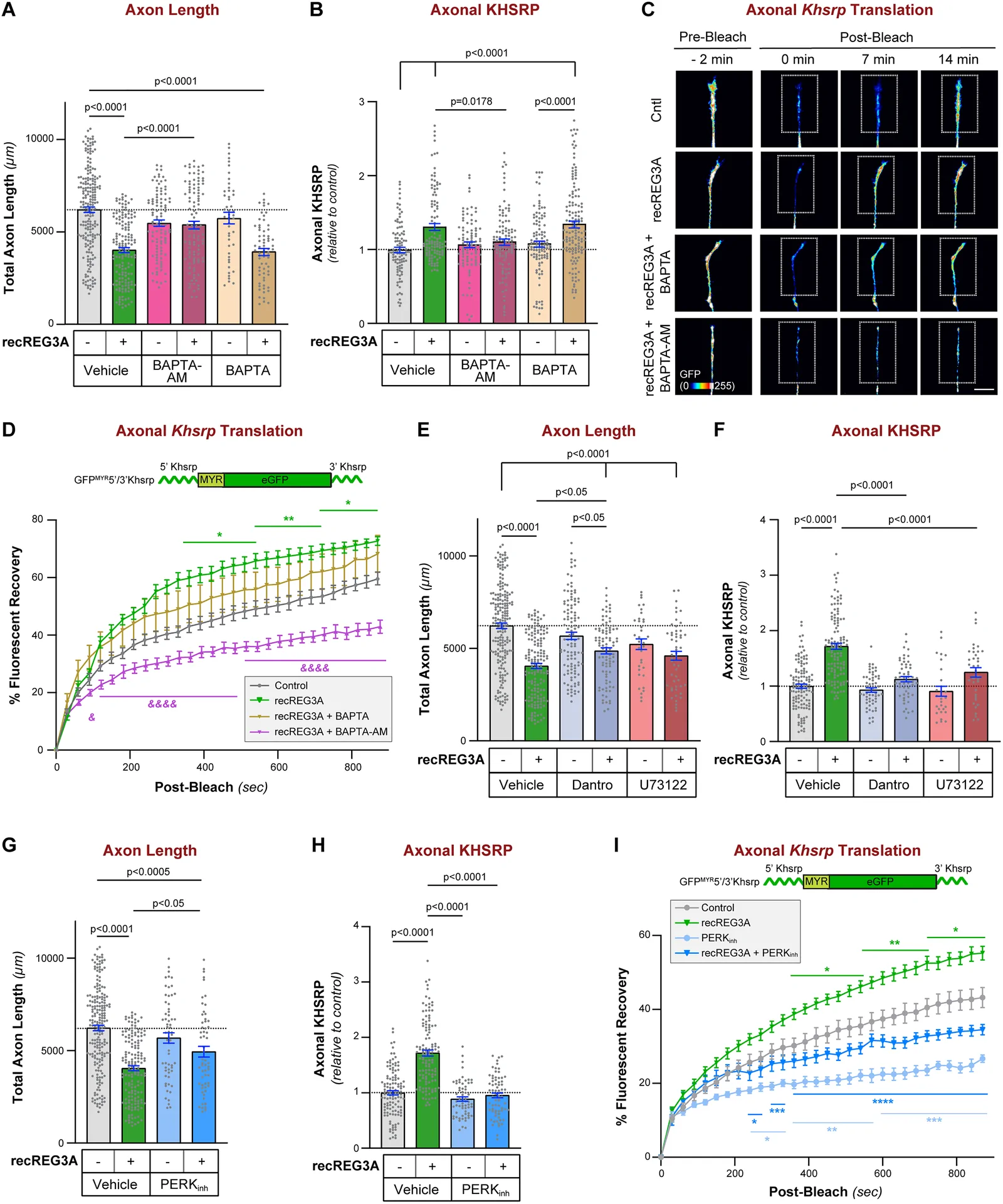
Axonal REG3A signaling requires release of intracellular Ca^2+^ and an ER stress-like response. (**A** and **B**) Axon growth (A) and axonal KHSRP levels (B) for DRG neurons ± recREG3A in presence of BAPTA-AM, BAPTA, or vehicle control (DMSO). One-way ANOVA with Tukey post-hoc analysis; mean ± SEM [*n* ≥ 46 in (A) and ≥ 99 in (B) across ≥3 replicate cultures]. (**C** and **D**) Axonal FRAP analyses for GFP^MYR^5’/3’Khsrp reporter in DRG neurons treated as in (A). Image sequences shown in (C) and quantitation in (D). Two-way ANOVA with Sidak post-hoc analysis; mean ± SEM; *P ≤ 0.05 and **P ≤ 0.005 vs. control and &*P* ≤ 0.05 and &&&&*P* ≤ 0.001 vs. RecREG3A (*n* ≥ 10 axons across ≥3 replicate cultures); scale bar, 10 μm. (**E** and **F**) Axon growth (E) and axonal KHSRP levels (F) for DRG neurons ± recREG3A in presence of dantrolene or U73122. One-way ANOVA with Tukey post-hoc analysis, mean ± SEM, [*n* ≥ 36 neurons in (E) and ≥ 28 in (F) across ≥3 replicate cultures]. (**G** and **H**) Axon growth (G) and axonal KHSRP levels (H) for DRG neurons ± recREG3A in presence of PERK inhibitor (PERK_inh_). One-way ANOVA with Tukey post-hoc analysis, mean ± SEM [*n* ≥ 60 in (A) and ≥ 34 in (B) across ≥3 replicate cultures]. (**I**) FRAP analysis for axonal GFP^MYR^5’/3’Khsrp transfected adult DRG cultures treated as in (G). Two-way ANOVA with Tukey post-hoc analysis, mean ± SEM; *P ≤ 0.05, **P ≤ 0.005, ***P ≤ 0.0005, ****P < 0.0001 (*n *≥ 10 axons across at least three culture preparations with colors matched to condition vs. control).

Since *Khsrp* mRNA translation increases with elevated Ca^2+^ and recREG3A increases axonal *Khsrp* translation, we asked if REG3A regulates Ca^2+^ entry into or intracellular release within axons. Chelating intracellular but not extracellular Ca^2+^ (using BAPTA-AM vs. BAPTA, respectively) blocked the effects of recREG3A on axon outgrowth, axon branching and axonal KHSRP elevation in DRG cultures (Fig. 4, A and B, and fig. S3F). By FRAP analyses the recREG3A-dependent increase in axonal translation of *GFP^MYR^5’/3’Khsrp* was also significantly attenuated by intracellular but not extracellular Ca^2+^ chelation (Fig. 4, C and D). Thus, REG3A activates release of intracellular Ca^2+^ stores to increase axonal *Khsrp* translation and reduce axon growth.

While ER, mitochondria, and lysosomes are potential sources for Ca^2+^ store release in axons, we had previously found that axonal *Khsrp* translation requires activation of PERK with subsequent phosphorylation of the translation factor eIF2α (*4*), a pathway that is activated upon ER Ca^2+^ depletion (*22*). Thus, we asked if inhibition of ER Ca^2+^ channels would impact responses to recREG3A. Inhibition of the ER Ryanodine receptor (RYR) with dantrolene or prevention of ER inositol phosphate-3 receptor (IP3R) activation by inhibition of phospholipase C (PLC) with U73122 each partially reversed recREG3A’s axon growth attenuation and axonal KHSRP elevation (Fig. 4, E and F, and fig. S3G), suggesting that both RyR and IP3R activation lie downstream of REG3A. Consistent with REG3A-dependent ER Ca^2+^ release, inhibiting PERK (with GSK2606414) also prevented the reduction in axon growth, increase in axon branching, and increase in axonal KHSRP seen with recREG3A treatment (Fig. 4, G and H, and fig. S3H). RecREG3A dependent axonal translation of *GFP^MYR^5’/3’Khsrp* was also prevented by PERK inhibition (Fig. 3I). Furthermore, the Integrated Stress Response Inhibitor (ISRIB), which prevents the integrated stress response by activating the translation factor eIF2B (*23*), prevented the REG3A-driven increase in axonal KHSRP (fig. S3I). Finally, recREG3A stimulation increased axonal eIF2α^PS51^ and this was prevented by PERK inhibition (fig. S4, A to C). Taken together, these data indicate that recREG3A slows axon growth by stimulating Ca^2+^ release from ER stores to activate PERK→eIF2α → *Khsrp* translation pathway.

### Endogenous REG3A decreases axon growth by episodically elevating growth cone Ca^2+^ in cultured neurons

**Fig. 5.**
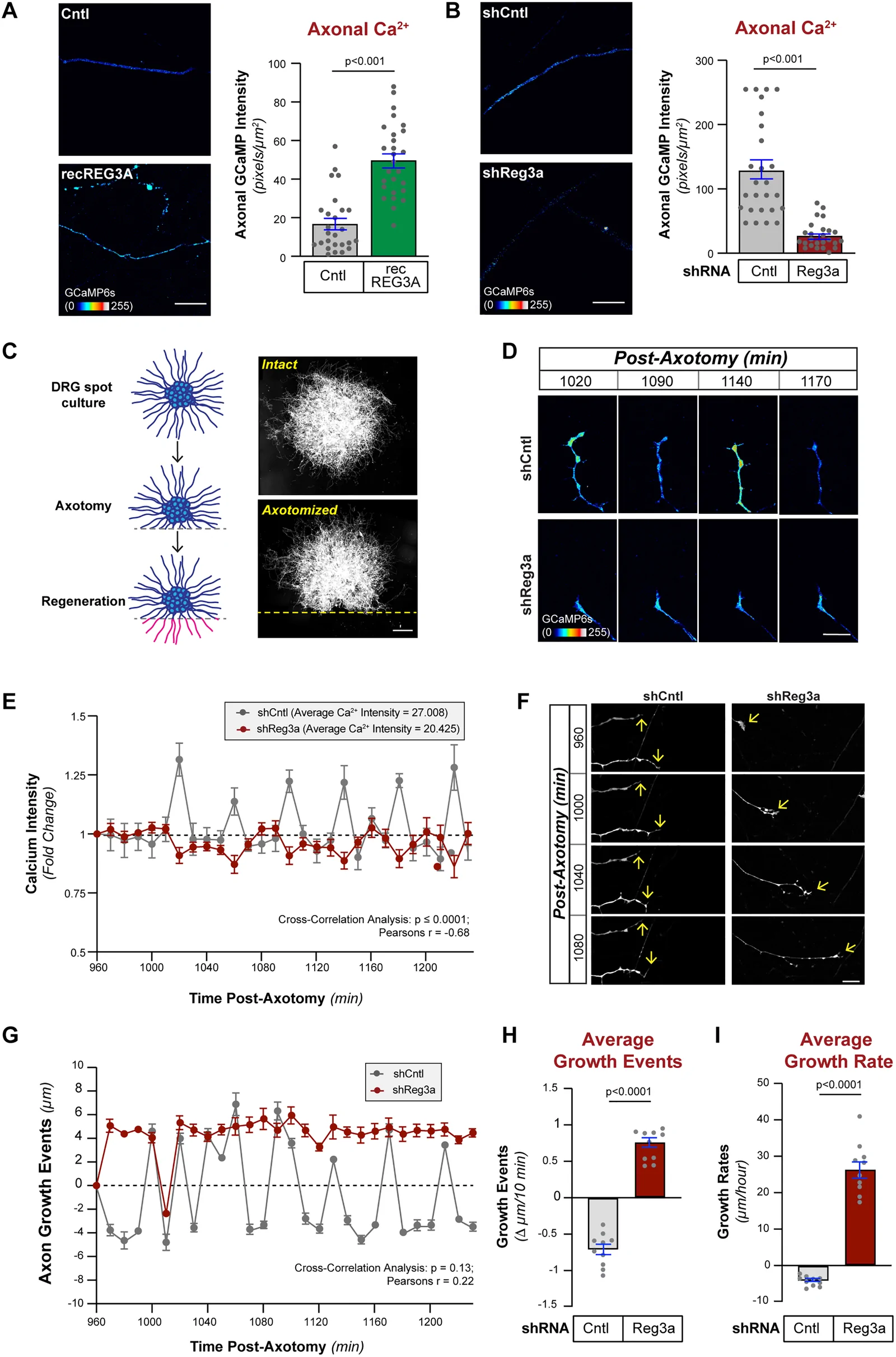
Endogenous REG3A attenuates axon growth by increasing axoplasmic Ca^2+^ and activating KHSRP synthesis in axons. (**A** and **B**) Exposure matched images of intact axons (left) and quantitation (right) for DRGs transduced with AAV-GCaMP6s ± 1 hr. recREG3A (A). GCaMP6s images of intact axons (left) and quantitation (right) for DRG cultures co-transduced with AAV-shRNAs and -GCaMPS6s (B). Imaging parameters for (A) and (B) were set for recREG3A or shReg3A conditions with control parameters matched within each experiment. Student’s *t* test, mean ± SEM (*n *≥ 75 neurons over ≥3 culture preparations); scale bar, 10 μm. (**C**) Schematic for in vitro axotomy in DRG spot cultures with representative images; scale bar, 100 μm. (**D** and **E**) GCaMP6s signals in distal axons of shCntl vs. -hReg3a transduced DRG spot cultures; image sequences (D) and quantitation of mean signal intensities ± SEM (E). Mean of absolute GCaMP6s signals over the entire imaging sequence for both are shown in the legend. *P* value by cross-correlation analysis; *n* = 10 axons across 4 separate culture preparations; scale bar, 10 μm. (**F** and **G**) Axon growth events for neurons in (D) based on BFP signals from the AAV-shRNAs. Image sequences (F) and quantitation of axon growth events (G) as mean change ± SEM shown. *P* values by cross-correlation analyses (*n* = 10 axons across 4 separate culture preparations); scale bar, 10 μm. (**H** and **I**) Quantitation of the growth events (H) and growth rates (I) across the imaging sequences in (G) as mean ± SEM. *P* values by Student’s *t* test (*n* = 10 axons across 4 separate culture preparations).

The findings above provided circumstantial but not direct evidence for elevated axoplasmic Ca^2+^ as a key regulator of REG3A’s effects on axons, so we asked whether recREG3A increases Ca^2+^ levels in axons of cultured DRG neurons. DRG cultures transduced with AAV expressing the genetically-encoded Ca^2+^ indicator GCaMP6s showed a clear increase in axonal Ca^2+^ signals after recREG3A stimulation (Fig. 5A). Co-transduction with AAV-GCaMP6s plus AAV-shReg3a or -shCntl showed a decrease in basal axonal GCaMP6s signals in shReg3a- compared to shCntl-transduced DRG cultures (Fig. 5B). Axonal signals for the Fluro-4 Ca^2+^ indicator were similarly increased after recREG3A treatment and decreased with *Reg3a* depletion (fig. S4, D and E). Thus, REG3A increases axoplasmic Ca^2+^ levels.

We next used a DRG ‘spot culture’ technique, where axons are physically and visually isolated from their soma; this allowed us to visualize growing axons as well as regeneration of in vitro transected axons (Fig. 5C). Characterizing growing intact axons in the DRG spot cultures showed increase in both axonal eIF2α^PS51^ and KHSRP after recREG3A treatment (fig. S4, F and G). To analyze regenerating axons, axons shafts were manually transected and then distal axons proximal to the transection site were visualized 16 h later to allow the initial Ca^2+^ increase in severed axons to subside and regeneration to fully initiate. 16 h after manual axon transection, regenerating axons in these DRG spot cultures showed decreased axonal eIF2α^PS51^ and KHSRP in cultures when endogenous *Reg3a* was depleted (fig S4, H and I). We next used DRG spot cultures that were co-transduced with AAV-GCaMP6s + AAV-shReg3a or AAV-GCaMP6s + AAV-shCntl to determine if Ca^2+^ levels dynamically change over time in regenerating axons. Average growth cone GCaMP6s signals were significantly lower in the shReg3a- vs. shCntl-transduced neurons beginning at 16 h post axotomy (fig. S4J). Fold-changes in axoplasmic Ca^2+^ for each time point relative to the average growth cone GCaMP6s signals across the entire time sequence showed varying growth cone Ca^2+^ signals in shCntl-transduced cultures, with sharp increases approximately every 30–70 min (Fig. 5, D and E). In contrast, shReg3a-transduced cultures showed significantly less variability in growth cone Ca^2+^ over the imaging sequence (Fig. 5, D and E). We assessed axon growth events in these same cultures based on the BFP included in the shCntl and shReg3a RNA vectors. Axons of the shCntl-transduced neurons showed alternating extension and retraction events, while the shReg3a-transduced neurons showed overall processive axon growth (Fig. 5, F and G, and movie S1). Average directionality of axon growth events and overall growth rates were significantly increased in the *Reg3a*-depleted DRG cultures (Fig. 5, H and I). Thus, depletion of endogenous *Reg3a* decreases and stabilizes growth cone Ca^2+^ and increases axon growth.

### Reciprocally oscillating synthesis of KHSRP and REG3A in regenerating axons of cultured neurons

**Fig. 6.**
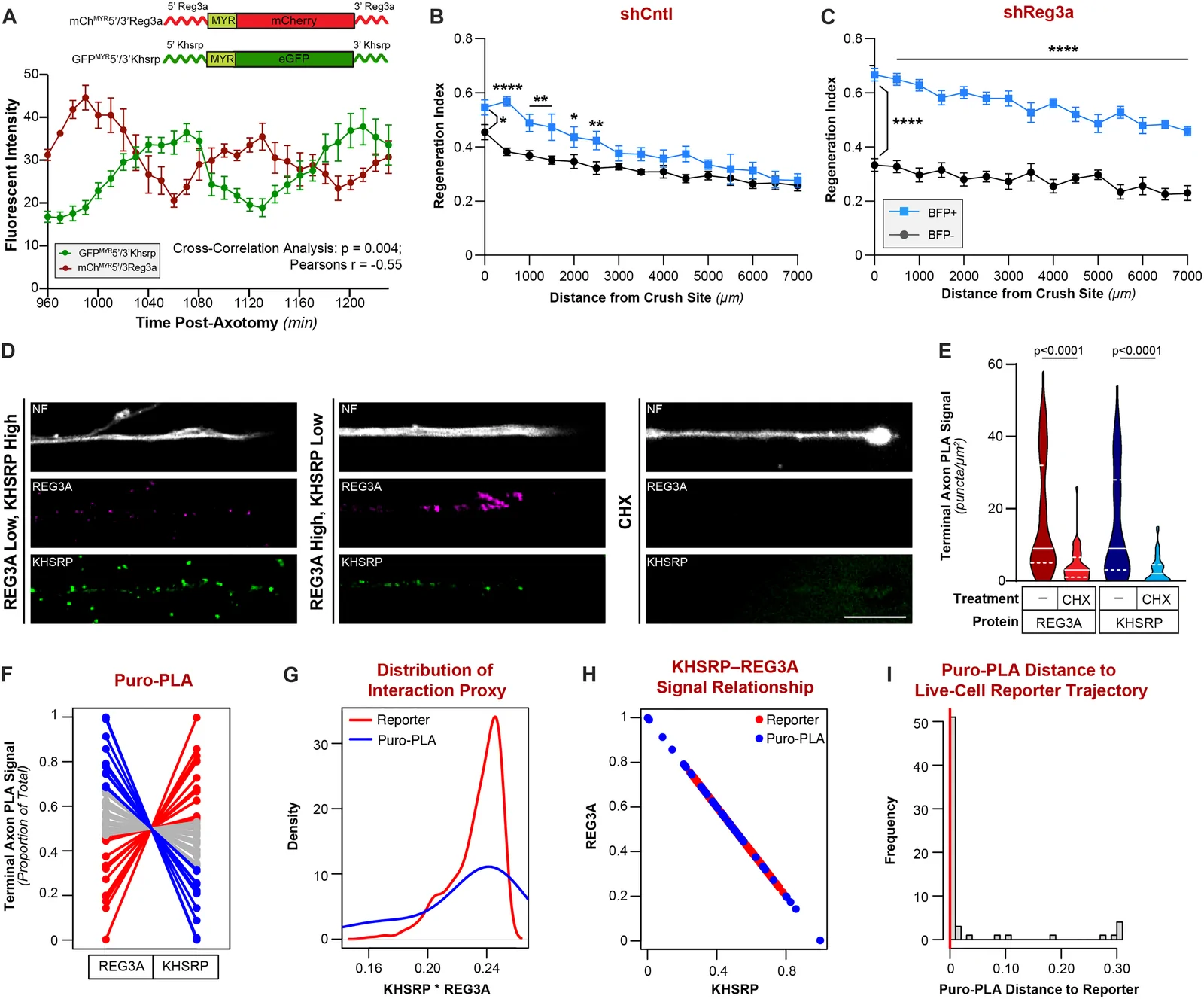
Axon-intrinsic reciprocal regulation of KHSRP and REG3A synthesis. (**A**) Growth cone signals for GFP^MYR^5’/3’Khsrp and mCh^MYR^5’/3’Reg3a in proximal severed axons in DRG spot cultures over 16–20.5 h post-axotomy imaging sequences. P value by cross-correlation analyses, mean ± SEM (*n* = 10 axons across 4 separate culture preparations). (**B** and **C**) Regeneration indices of BFP positive vs. negative regenerating axons of shCntl- (B) and shReg3a-transduced (C) mice. Two-way ANOVA with Sidak post-hoc analysis, mean ± SEM; *P ≤ 0.05, **P ≤ 0.005, ***P ≤ 0.0005, and ****P < 0.0001 (*n* ≥ 5 mice per group). (**D** and **E**) REG3A and KHSRP Puro-PLA images (D) and quantitation (E) for regenerating axons (18 h post axotomy) in CHX vs. vehicle treated DRG spot cultures. *t* test, mean ± SEM; scale bar, 10 μm. (**F**) Puro-PLA axon signals stratified by KHSRP/REG3A ratio. Signals are stratified into low KHSRP/REG3A ratio quartile groups based on the log-ratio distributions median. *P* ≤ 0.001 for high REG3A + low KHSRP vs. median and low REG3A + high KHSRP vs. median based on Wilcoxon sum-test. (**G**) Kernel density estimates of KHSRP × REG3A show distinct distributions between conditions. Live-cell signals from (A) exhibit a narrower, higher-intensity peak, while Puro-PLA signals from (F) display a broader distribution, indicating increased variability in co-occurrence. (**H**) Scatterplot of Puro-PLA and reporter signals for individual axons reveals a strong inverse relationship, with both occupying a shared linear trajectory. This indicates that the signals from the two approaches are tightly coupled and constrained along a low-dimensional manifold. (**I**) Histogram of distances from static cells to the live-defined trajectory. Most cells cluster near zero, indicating that they conform to the coordinated relationship. A subset of cells exhibits larger distances, forming a long tail that represents deviation from the trajectory.

Interestingly, regenerating axons in DRG spot cultures co-transfected with translation reporters showed that fluorescence of GFP^MYR^5’/3’Khsrp and mCh^MYR^5’/3’Reg3a translation reporters exhibited peaks and troughs with opposing periodicity (Fig. 6A). Individual axons varied substantially, so these analyses required that the x-axes for individual axons be aligned for an initial peak in the *Reg3a* reporter fluorescence. This variability raised the possibility of an autocrine rather than paracrine response to endogenous REG3A. Consistent with this possibility, adjacent axons in the same field of view did not show synchrony for the GFP^MYR^5’/3’Khsrp and mCh^MYR^5’/3’Reg3a reporter signals (fig. S5, A and B). Furthermore, the BFP negative axons of non-transduced neurons in the nerves of the AAV-shReg3a-BFP transduced mice from Fig. 2, A and B, showed regeneration indices similar to the BFP positive axons in the AAV-shCntl-GFP-transduced mice (Fig. 6, B and C).

To further test the possibility for reciprocal regulation of *Khsrp* and *Reg3a* mRNA translation in growth cones, we employed a multiplex puromycinylation combined with proximity ligation assay (Puro-PLA) to simultaneously visualize endogenous KHSRP and REG3A translation products in individual axons at 18 h after transection. CHX treatment showed that the KHSRP and REG3A Puro-PLA signals in distal axons seen with a 15 min puromycin exposure are from newly synthesized proteins (Fig. 6, D and E). Without CHX treatment, there was substantial variation in the KHSRP and REG3A Puro-PLA signals of individual growth cones but comparing the ratio of KHSRP:REG3A Puro-PLA signals in individual growth cones showed an overall inverse correlation for translation of the two proteins (Fig. 6, D and F, and fig. S6A). Notably, non-neuronal cells in these cultures showed robust KHSRP Puro-PLA puncta, as expected from previous studies (*4*), but Puro-PLA signals for REG3A were overall similar to the REG3A in the CHX-treated cultures (fig. S5C); this indicates that REG3A synthesis is much higher in axons than in non-neuronal cells in the DRG cultures.

We next tested whether the variability seen in the single time-point Puro-PLA signal intensities across individual axons represents a static view of the translation reporter signals for GFP^MYR^5’/3’Khsrp and mCh^MYR^5’/3’Reg3a from individual axons over time. Multivariate 2-sample E-test of equal distributions gave an E-statistic = 2.7514, *P* value = 0.1738 at 1000 repeated analyses, which suggests similarity between the 2 data sets. Normalized per-axon signal intensities for KHSRP and REG3A were extracted for both reporter and Puro-PLA analyses and the relationship between signals were plotted directly in signal space (fig. S6, B and C); an interaction proxy was defined as the product of the two signals (KHSRP × REG3A), representing their co-occurrence within individual axons (Fig. 6G). As a more quantitative measure of similarity, a reference trajectory describing the coordinated relationship between live-cell GFP^MYR^5’/3’Khsrp and mCh^MYR^5’/3’Reg3a signals was defined by fitting a locally weighted regression (lowess) curve in signal space; using the 95th percentile of the GFP^MYR^5’/3’Khsrp and mCh^MYR^5’/3’Reg3a signal trajectory deviation as a threshold, 84.3% of Puro-PLA signals for KHSRP and REG3A signals fall within the live-cell imaging interaction manifold for the axonal GFP^MYR^5’/3’Khsrp and mCh^MYR^5’/3’Reg3a signals (Fig. 6, H and I, and fig. S6, D and E). Thus, the static images for axonal Puro-PLA largely recapitulate the live interaction manifold for GFP^MYR^5’/3’Khsrp and mCh^MYR^5’/3’Reg3a but also include a subpopulation with significantly increased deviation, consistent with incomplete coordination between KHSRP and REG3A in the two approaches.

### REG3A is secreted and concentrates along the axon

The analyses of individual axons above points to an autocrine effect of axonally-derived REG3A. For such to occur, REG3A would need to be secreted from axons. Thus, we used confocal imaging to assess REG3A localization in cultured DRG neurons and sciatic nerves. As expected, immunofluorescent signals for endogenous REG3A protein were increased in cultures of L3–5 DRGs from mice that had previously undergone an in vivo sciatic nerve crush for ‘injury-conditioning (*15*) and in regenerating sciatic nerve compared to naïve DRG cultures and uninjured nerve, respectively (fig. S5D). In both the injury-conditioned DRG cultures and regenerating sciatic nerve, the REG3A signals appeared to be concentrated along the periphery of the axons by orthogonal projections of confocal XYZ image stacks (fig. S5, D and E, and movies S2 and S3). The striking increase in REG3A immunoreactivity was confirmed in teased sciatic nerve preparations, where REG3A signals were clearly seen traversing the node of Ranvier marked by Ankyrin G immunostaining (fig. S5F). Axons have functional equivalents of ER and Golgi apparatus for secretion of newly synthesized proteins (*24*). Lectin proteins bind to proteoglycans (*25*), so it is conceivable that secreted REG3A protein adheres to the membrane and juxta-membrane region. Consistent with this, heparinase treatment of DRG cultures prior to immunofluorescent staining drastically decreased the signals for REG3A in the injury-conditioned DRGs (fig. S5D and movie S2). Furthermore, immunoblots from DRG cultures showed that REG3A protein in both lysates and insoluble debris from RIPA buffer lysis, with much more in the insoluble pellet that could be consistent with membrane adherence (fig. S5G). Although we cannot fully exclude non-neuronal contributions to the increased REG3A, lack of REG3A Puro-PLA signals in Schwann cells in the DRG cultures above (fig. S5C) and lack of *Reg3a* mRNA in Schwann cell populations of previously published single cell RNA sequencing data sets for injured sciatic nerve (*26*), combined with commensurate increase in *Reg3a* mRNA in L3–5 DRGs and sciatic nerve axons 2 d after sciatic nerve crush (Fig. 1A and fig. S1, I and J), all point to an axonal origin for the extracellular REG3A protein in the proximal sciatic nerve after injury. Taken together, these data suggest that axonal synthesized and secreted REG3A signals in an autocrine fashion to slow axon growth through Ca^2+^ store release-dependent translation of *Khsrp* mRNA.

## DISCUSSION

**Fig. 7.**
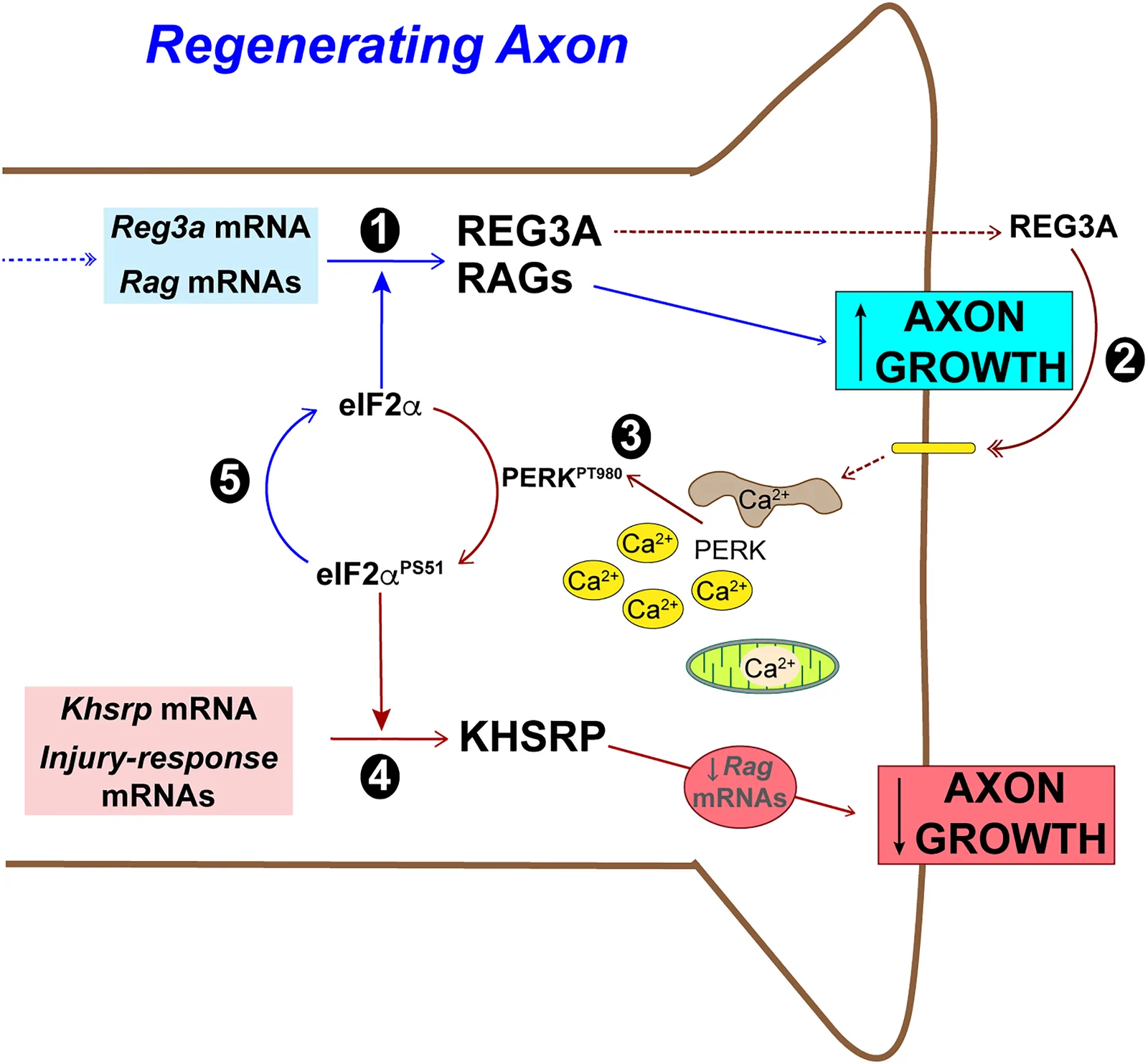
Mechanism for attenuation of axon growth by REG3A to KHSRP signaling. Schematic for axon-intrinsic translational regulation of *Khsrp* mRNA by axonally synthesized REG3A in regenerating axons. (1) Injury increases cell body *Reg3a* mRNA that is transported into regenerating axons and translated under low Ca^2+^ levels when eIF2α is not phosphorylated. (2–3) Nascent REG3A secreted from the distal axon activates a transmembrane signal for ER Ca^2+^ release activating PERK→eIF2α^PS51^. (4) Increased eIF2α^PS51^ activates translation of *Khsrp* mRNA promoting RAG mRNA decay. (5) Subsequent dephosphorylation of eIF2α promotes translation of *Reg3a* to generate an oscillating translation of *Khsrp* and *Reg3a*.

Interventions that can accelerate axon regeneration have great potential to increase recovery from PNS nerve injuries, and our data point to an axon-intrinsic REG3A → Ca^2+^ → KHSRP pathway as a target for accelerating nerve regeneration (Fig. 7). Many lines of evidence indicate that axonally synthesized proteins provide an initial response to injury and then later promote regeneration of the injured axons through local actions of the newly synthesized proteins (*27*, *28*). While most of the axonally-synthesized proteins studied to date promote axon growth or neuronal survival after injury, axonal translation of *Khsrp* impedes axon growth by promoting decay of ARE-containing axonal mRNAs (*4*). *Khsrp* translation is increased by axoplasmic Ca^2+^ elevation following injury, but its protein product stays elevated in axons well beyond the initial axotomy-induced Ca^2+^ elevation subsides (*4*). Here, we show that axonally-synthesized REG3A protein provides a means to intermittently activate *Khsrp* translation in regenerating axons. Consequently, axonal translation of *Reg3a* slows PNS nerve regeneration. Our findings suggest the following sequence of events as summarized in Fig. 7: *Reg3a* mRNA is transported into PNS axons after injury, the encoded REG3A is synthesized in and then secreted from axons, REG3A activates an intra-axonal signal that culminates in ER Ca^2+^ release and eIF2α phosphorylation, resulting in increased translation of axonal *Khsrp* and growth slowing through KHSRP’s RNA decay effects, including decay of regeneration associated gene mRNAs [e.g., *Gap43*, *Snap25*, and *Prenyl-Cdc42* (*4*, *6*)]. Taken together, our findings bring new knowledge for the functions of axonally-synthesized proteins and emphasize that regenerating axons experience an intrinsic stress response through intermittent translation of *Reg3a,* REG3A-dependent ER-Ca^2+^ release, and subsequent activation of PERK→eIF2α → *Khsrp* translation.

REG3A is a member of a family of lectin-like proteins that include REG3B and REG3G. Murine REG3A is about 59% identical to REG3B and REG3G proteins. REG3 proteins have been functionally linked to regeneration of pancreatic islet cells and REG3A has bactericidal activity in the intestine (*9*). EXTL3 protein was reported as a cell surface receptor for REG3 proteins (*29*). Several inflammatory cytokines have been reported to increase REG3A expression in epithelial systems where REG3A binding to EXTL3 activates the phosphatidyl-inositol-3 kinase (PI3K) to AKT signaling pathway (*30*). REG3G has also been reported to activate PI3K-AKT as well as the RAS/RAF-ERK signaling pathways through EXTL3 binding (*10*–*12*). Though EXTL3 has well-established roles as a glycosyl-transferase in the Golgi apparatus (*31*), cell surface localization of EXTL3 has been reported for hippocampal neurons, N2a cells and neuron-like PC12 cells (*13*). Further, REG1α protein, which shares less homology to REG3A than the REG3 family members (42% sequence identity), was reported to promote neurite growth in PC12 cells by binding to EXTL3 (*13*). PI3K, AKT, Ras/Raf and ERK signaling are typically growth-promoting in neuronal contexts rather than the clear attenuation of growth that we see with both recombinant and endogenous REG3A in the experiments here. REG3B was also shown to activate JAK/STAT3 signaling in carcinoma cells (*32*), but STAT3 activation after injury promotes nerve regeneration and neuronal survival (*33*). Thus, REG3A’s attenuation of axon growth seems unlikely to occur through previously defined signaling events downstream of EXTL3 binding.

REG3A’s function as a bactericide is linked to its glycoprotein-binding activity, with hexamers of REG3A protein dimers permeabilizing the bacterial membrane (*34*, *35*). Cell membrane permeabilization by a naturally occurring peptide fragment of REG3A (termed PAP) has also been reported to stimulate pancreatic beta-cell proliferation (*36*). Pore-forming activities reported for REG3A could promote Ca^2+^ entry after REG3A secretion or recREG3A exposure, but we find that neither the axon growth attenuation nor the axonal KHSRP elevation by REG3A was affected by depletion of extracellular Ca^2+^. Activation of PERK and subsequent eIF2α phosphorylation increasing axonal KHSRP synthesis is consistent with release of ER Ca^2+^ stores in response to both recombinant and endogenous REG3A. Though Ca^2+^ influx can trigger subsequent ER Ca^2+^ release (*37*), extracellular Ca^2+^ chelation did not prevent effects of recREG3A on DRG axons, so REG3A-dependent Ca^2+^ entry cannot explain the effects of REG3A seen here. Taken together, our data argue that the effects of axonally-synthesized REG3A are through a different receptor and signaling mechanism than previously published for REG3A. Further, our data suggest that this is an autocrine, axon-intrinsic mechanism that slows axon growth.

Elevated axoplasmic Ca^2+^ levels play a key role in mounting a local response to neural injury. Extracellular Ca^2+^ is required to seal severed neurites in cultures of differentiated neuronal-like PC12 cells (*38*), and increased Ca^2+^ is needed for growth cone formation after axon severing in cultured *Aplysia* neurons (*39*). Growth cone formation after injury in cultured neurons requires axonal protein synthesis in cultured rodent DRG neurons (*40*). In mammalian systems, axotomy triggers a retrograde Ca^2+^ wave that causes initial changes in neuronal gene expression needed for activating regeneration-associated gene expression (*41*). Those gene expression programs are further modified by retrograde transport of axonally synthesized proteins, including Importin β1, STAT3α, PPARγ and Luman/CREB3 (*33*, *42*–*44*). Injury-induced Ca^2+^ elevation is needed for the initial synthesis of several of these injury-response proteins in axons. Axonal syntheses of Luman/CREB3 and CALR have been linked to the unfolded protein or integrated stress response in axons (*21*, *45*). Indeed, local increase in eIF2α^PS51^ levels, as we show here that REG3A causes, have consistently been shown to impact translation of specific mRNAs in axons (*7*, *20*, *44*–*46*). Interestingly, we see that recREG3A increases axonal levels of CALR protein, whose mRNA is translated in axons by elevated Ca^2+^ and PERK activation (*7*, *20*, *21*). Inflammation and injury have been reported to increase REG3A expression in rodent sensory neurons within 2 d following the inflammation or injury (*47*). Although the authors did not address function of the neuronal REG3A, they concluded that increased peripheral nerve REG3A levels derive from anterograde transport based on nerve ligation studies (*47*). Axonal localization of *Reg3a*, combined with localized translation of an axonal reporter mRNA with *Reg3a*’s 5′ and 3’UTRs indicate that the increases in axonal REG3A protein after axotomy derive from anterograde mRNA transport and subsequent translation in axons.

The reciprocally oscillating axonal signals for the translation reporters GFP^MYR^5’/3’Khsrp and mCh^MYR^5’/3’Reg3a suggest that translation of *Reg3a* generates increased ER Ca^2+^ release to activate *Khsrp* translation and slow axon growth through an axon-intrinsic autocrine signaling mechanism. Although not a perfect overlap, the 83.5% correlation between the live cell translation reporter data and static Puro-PLA data for axons supports the conclusion that endogenous REG3A and KHSRP synthesis oscillates in individual axons. The outliers in this comparison could result from varying sensitivity of the live-cell reporter imaging and Puro-PLA approaches, or it could represent deviation of individual axons. Nonetheless, locally cycling eIF2α between its non-phosphorylated and phosphorylated states in distal axons would provide a mechanism to drive this translational specificity. Based on previous findings, axons have the machinery necessary to phosphorylate and dephosphorylate eIF2α^S51^ (*4*, *7*, *21*). Thus, the findings reported here suggest that targeting the signaling mechanisms underlying REG3A to KHSRP signaling can bring new strategies to accelerate nerve regeneration.

## MATERIALS AND METHODS

### Key reagents and resources

Table S1 summarizes key reagents utilized in these studies and the sources of those reagents where indicated.

### Animal use

All animal use was approved by the Institutional Animal Care and Use Committee of the University of South Carolina under protocols 2789–102080-120251 or 2741–101976-120624. Adult (8–16 wks old) male and female *Khsrp* knockout (*Khsrp*^−/−^) (*48*) and wildtype (C57Bl/6) mice were used for all experiments. Isoflurane inhalation was used for anesthesia in all survival surgery experiments (see below) and animals were euthanized by CO_2_ asphyxiation as indicated for results.

For peripheral nerve injury, 8–12 week old, anesthetized mice were subjected to sciatic nerve crush at mid-thigh level as previous described (*4*). Briefly, the sciatic nerve was exposed and then crushed using #2 fine jeweler’s forceps twice for 15 sec each, ∼1.5–2 cm from its origin, proximal to the trifurcation. Axotomy was monitored by the initial contraction of the hind limb upon application of pressure to the sciatic nerve and then the lack of hind paw extension during and upon recovery from anesthesia. For animals undergoing unilateral nerve crush, the contralateral nerve was exposed but not crushed (i.e., ‘sham’ control).

We have previously shown that nerve injected AAV is retrogradely transported to the neuronal cell bodies whose axons transverse the nerve resulting in a neuronal specific transduction (*4*). For shRNA mediated knockdown experiments 4 μl of 5 x 10^11^ particles of AAV9-TagBFP2-U6-shReg3a or TagBFP2-U6-scramble-shRNA (Vector Builder, Chicago, IL) were diluted in 600 mM NaCl (total volume = 5 μl) and injected into the proximal sciatic nerve at ∼1.2–1.5 cm from origin. 14 d after viral transduction, animals were subjected to a bilateral sciatic nerve crush at ∼0.5 cm distal to injection site on both the left and right sciatic nerve as above. For consistency between animals, a single experimenter performed the viral infections and crush injuries within each series of animals.

### Cell culture

Adult mouse dorsal root ganglia (DRG) were harvested and placed in ice-cold Hibernate-A medium (BrainBits). For experiments with naïve DRG neurons, all DRGs including lumbar, thoracic and lower cervical were collected. For in vivo injury conditioning, only lumber segment 3–5 DRGs were collected. DRGs were rinsed five times with DMEM/F12 + 10 U/ml Pen/Strep (CAT#15–140-122, Fisher, Waltham, MA) and then ganglia were treated with 0.125 U/ml collagenase type II CAT#17–101-015, Fisher) in DMEM/F12 for 30 min at 37°C, 5% CO_2_. DRGs were triturated using a fire polished Pasteur pipette and pelleted by centrifuged at 100 xg for 10 min. DRGs were washed twice by resuspension with DMEM/F-12 + Pen/Strep and centrifugation.

Following washes, cells were resuspended in DMEM/F-12 media supplemented with 10% fetal bovine serum (Gibco, #10437028) 10 μM cytosine arabinoside (Sigma, St Louis, MO; # C6645), 1x L-glutamine (Fisher; # A2916801), 1x N1 medium supplement (0.5 mg/ml Insulin (Sigma; # I9278), 0.5 mg/ml Sodium Selenite (Sigma; # S5261), 0.5 mg/ml Transferrin (Sigma; #T8158), 1.6 mg/ml Putrescine (Spectrum; # P1834), 0.73 μg/ml Progesterone (Spectrum; # P1834), in Earle’s Buffered Salt solution, no phenol red (Thermo Scientific, AAJ67559AE). Dissociated DRGs were plated immediately or transfected and then plated (see below) on poly-L-lysine/laminin-coated glass substrates. For coating substrates, coverslips were incubated in 50 μg/ml poly-L-lysine (Fisher; #A005C) for 2 h at 37°C and 5 μg/ml laminin (Sigma) at 4°C overnight. 12 mm glass coverslips, 35 mm black wall glass bottom dishes (WillCo Wells from Ted Paella, Redding, CA; # HBSB-3522), or chambered coverslips (Ibidi, Gräfelfing, Germany; # 80297) were used.

For DRG ‘spot cultures’, isolated ganglia were treated with collagenase as above for 30 min at 37°C, 5% CO_2_. DRGs were then triturated 10–15 times with a 1000 μl pipette tip followed by a second incubation at 37°C, 5% CO_2_ for 10 min. Ganglia were dissociated into single cell suspension using a 1000 μl pipette tip in 4 ml of media containing 1x penicillin/streptomycin solution. Dissociated ganglia were centrifuged for 10 min at 700 xg. Cell pellet was resuspended in fresh DRG culture medium and plated at a density of approximately 2.8 DRGs per 7 μl (up to 45 DRGs harvested per mouse). For this, dissociated ganglia were gently triturated 10 times with 20 μl pipette tip and 7 μl placed onto poly-D-lysine/laminin-coated chambered coverslips at up to 2 well-separated ‘spots’ per well. Spotted DRGs were incubated at 37°C. 5% CO_2_ for 7 min and then DRG culture medium was added along the wall of the culture vessel adequate to fully cover the cells and not be at risk for evaporation. These ‘spot cultures’ were incubated at 37°C, 5% CO_2_ for 7–8 d. For medium changes, half volume of the medium in each well was removed and replenished with fresh DRG culture medium at d 1 and 5 post-dissociation. To axotomize the DRG spot cultures for in vitro regeneration assay, axons were cut on one side of the spot under a stereomicroscope using a sterilized 0.6 mm thick x 2.75 mm wide flat carbon steel surgical blade (Fine Science Tools, 10035–10). Axons were cut at distance approximately equal to the radius of the spot (i.e., approximately 1 mm from the closest soma). Axon regrowth from the injury site was evaluated at 16–30 h post-axotomy as indicated in the results section.

For transducing spot cultures with AAV9, dissociated ganglia resuspended in DRG media were pelleted by centrifugation at 700 xg for 10 min. The pellet was resuspended in fresh DRG culture media containing 5 μl of 5 x 10^11^ particles AAV9shReg3a-TagBFP2 or AAV9-shControl-TagBFP2 virus.

For cDNA transfections, dissociated ganglia were pelleted after washing in DMEM/F-12 at 100 x g for 5 min and then resuspended in 100 μl ‘nucleofector solution’ for Rat Neuron Nucleofector kit (Lonza, Alpharetta, GA; # VPG-1003) or 20 μl solution for Small Cell Number-SCN kit (Lonza; # VSPI-1003). 4–6 μg plasmid was electroporated for the Rat Neuron Kit and 1 μg for the Small Cell Number Kit using AMAXA Nucleofector apparatus (program G-013 for Rat Neuron kit and SCN-8 for SCN kit). Transfected DRGs were then plated as above.

For stimulation and inhibition in DRG cultures the following agents were used: recombinant human REG3A-His (277.8 nM; SinoBiological, Wayne, PA; # 11235-H08H), cycloheximide (150 μg/ml, Sigma), BAPTA-AM (3 μM; Sigma, St Louis, MO), BAPTA (3 μM; Cayman Chemical, Ann Arbor, MI), Thapsigargin (1 μM; Sigma), ISRIB (50 μM; Sigma), GSK260614 (90 μM; Bio-Techne Corp/Tocris, Minneapolis, MN), Dantrolene (10 μM, Sigma, # D9175), and U73122 (1 μM; Sigma, # 1268) 24 h after initial plating. Equal concentration of vehicle was used for inhibitors and chelators, and equivalent concentration of BSA was used for recREG3A. For chelators and inhibitors, cultures were incubated for 15 min prior to other manipulations. Cultures were then treated with recREG3A or control for 24 h followed by rinsing in 1x PBS and fixation in 4% paraformaldehyde in 1x PBS.

### Plasmid constructs and virus constructs

All fluorescent reporter constructs for analysis of RNA translation were based on eGFP with myristoylation element (GFP^myr^; originally provided by E. Schuman, Max Planck Institute) or mCherry plasmid with myristoylation element (mCh^myr^). Reporter construct containing 5′ and 3’UTR of *Khsrp* mRNA has been published (GFP^MYR^5’/3’Khsrp) (*4*). For Reg3a translation reporter (mCh^MYR^5’/3’Reg3a), the 5′ and 3’UTRs of mouse *Reg3a* were PCR-amplified and subcloned into mCh^myr^ reporter. pAAV-hSynapsin1-GCaMP6s-P2A-mRuby3 Ca^2+^ sensor plasmid was originally generated by L. Tian and was purchased from Addgene (*49*). AAV5 and AAV9 preparations were purchased directly from Addgene (112005-AAV5 and 112005-AAV9). AAV9-shCntl-BFP and -Reg3a-BFP were purchased from VectorBuilder.

### RNA isolation and analyses

RNA was isolated from cultured DRG neurons and sciatic nerves using the RNeasy Microisolation kit (Qiagen, Hilden, Germany). Dissociated DRG cultures were grown for 3 d, washed briefly with PBS then RNA was isolated following manufacturer’s protocol. Sciatic nerves were cut into small pieces and digested with collagenase at 37°C for 30 min with intermittent trituration. RNA was isolated from the collagenase-treated nerves using the RNAeasy Microisolation kit. RNA yield was quantified using fluorimetry with Ribogreen reagent (ThermoFisher, Waltham, MA) and 50 ng of RNA was reverse transcribed using the Sensifast cDNA synthesis kit (Bioline, London, UK). Droplet digital (dd) PCR products were detected using Evagreen reagent on a QX200 droplet reader (Biorad, Hercules, CA). Mitochondrial 12S RNA (*Mtrnr1*) mRNA levels were used for normalizing yields across different isolates as indicated in results. The following primers were used for ddPCR [all from Integrated DNA Technologies (IDT), Coralville, Iowa; all listed as 5′ to 3′]: *Mtrnr1*, sense – GGCTACACCTTGACCTAACG and antisense – CCTTACCCCTTCTCGCTAATTC; *Reg3a*, sense – TCTACAAGAGAGACAAGATGCTG and antisense – AGCTGGTACGTGGAGAGG.

### Single molecule fluorescence in-situ hybridization (smFISH)

smFISH plus immunofluorescence (IF) was used to detect *Khsrp* and *Reg3a* mRNAs in dissociated DRG cultures and sciatic nerve sections. Custom designed Cy3- and Cy5-labelled Stellaris probes (LGC Biosearch Tech, Middlesex, UK) for mouse *Khsrp* and *Reg3a* with Cy3- and Cy5-labelled scramble probes for control were used. Primary antibodies for smFISH/IF consisted of axon marker antibody containing mouse anti-β3-Tubulin (TUJ1, Biolegend; # 801201) and anti-Neurofilament marker (SMI312; Biolegend; # 837904) cocktail (1:200 for tissues and 1:500 for dissociated cultures) and chicken anti-GFP (1:200 for tissues and 1:500 for dissociated cultures; Abcam, Boston, MA; # ab13970). FITC-conjugated donkey anti-mouse and Alexa405-conjugated anti-chicken (1:500 each; Jackson ImmunoRes., West Grove, PA) were used as secondary antibodies.

For cultured neurons, smFISH/IF was performed as previously described with minor modifications (*8*). All steps were carried out at room temperature and all solutions were RNase free and made using DEPC-treated water unless otherwise noted. Coverslips were briefly rinsed in 1x PBS and then fixed in 2% PFA in 1x phosphate-buffered saline (1x PBS) for 15 min. Coverslips were rinsed 2 times in 1x PBS, then permeabilized in 0.3% Triton X-100 in 1x PBS for 10 min. Samples were prehybridized for 1 h in hybridization buffer (50% dextran sulphate, 10 μg/ml *E. coli* tRNA, 10 mM ribonucleoside vanadyl complex, 80 μg BSA, and 10% formamide in 2x SSC), and then incubated with 12.5 μM each Stellaris probe, TUJ1 and SMI312 cocktail (1:500 each), and chicken anti-GFP (1:500) in hybridization buffer for 16 h at 37°C. Coverslips were then washed in PBS + 0.3% Triton X-100 3 times, followed by incubation with FITC-conjugated donkey anti-mouse and Alexa405-conjugated donkey anti-chicken for 1 h. After rinsing in PBS, cells were post fixed in 2% PFA in 1x PBS for 15 min and washed 3 times with 1x PBS then DEPC-treated water. Coverslips were inverted and mounted on glass slides using Prolong Gold Antifade (ThermoFisher).

For sciatic nerve tissue, smFISH/IF was performed as previously described with minor modifications (*8*). All steps were carried out at room temperature and all solutions were RNase free and made using DEPC-treated water unless otherwise noted. Sciatic nerve segments were fixed overnight at 4°C in 4% PFA, washed with PBS then cryoprotected overnight in 30% buffered sucrose at 4°C. Nerves were cryosectioned at 20 μm thickness and stored at −20°C until used). Sections were brought to room temperature, washed three times in 1x PBS for 5 min each, and then incubated with 20 mM glycine followed by 0.25 M NaBH_4_ in PBS (3 times, 10 min each for both) to quench autofluorescence. Sections were quickly rinsed in 0.1 M Triethylamine (TEA) and then incubated in 0.1 M TEA +0.25% acetic anhydride for 10 min. After washing in 2x SSC, sections were permeabilized with 0.3% Triton X-100 in PBS for 10 min. Sections were rinsed with 1x PBS and incubated with 2x SSC + 10% formamide for 10 min. Sections were prehybridized for 1 h in hybridization buffer (50% dextran sulphate, 10 μg/ml *E. coli* tRNA, salmon sperm DNA, 10 mM ribonucleoside vanadyl complex, 80 μg BSA, and 10% formamide in 2x SSC), and then incubated with 12.5 μM each Stellaris probe in addition to TUJ1 and SMI312 cocktail (1:200 each), and chicken anti-GFP (1:200) in hybridization buffer for 16 h at 37°C. The following day, sections were washed in 2x SSC + 10% formamide at 37°C twice for 30 min each, followed by a 10 min incubation in 0.5x SSC at 37°C. Sections were briefly rinsed in 1x PBS + 1% Triton-X100 and incubated with FITC-conjugated donkey anti-mouse and Alexa405-conjugated donkey anti-chicken for 1 h. Sections were washed in three times in 1x PBS and DEPC-treated water, then mounted under glass coverslips with Prolong Gold.

smFISH and IF signals in tissue sections were imaged using Leica SP8X or Stellaris confocal microscope. 63x/NA 1.4 oil immersion objective and pulsed white light laser was used for imaging RNA in both culture and tissue samples. Scramble probe was used to set the maximum image acquisition parameters (laser power, HyD or PMT detector energy, & offset) that gave minimum signals with the control probes. XYZ image stacks were acquired across at least three separate locations in each section scanned nerve sections.

### Immunofluorescence (IF)

Standard IF was performed as previously described with all steps at room temperatures unless specified otherwise. Coverslips were fixed with 4% paraformaldehyde in 1 x PBS for 15 min at room temperature and washed 3 times in 1 x PBS. PBS washed neurons were permeabilized with 0.3% Triton X-100 in PBS for 15 min and then placed in block buffer [1X PBS + 10% Normal Donkey Serum (Jackson ImmunoRes)] for 1 h. Neurons were then incubated with primary antibodies diluted in block buffer overnight in a humidified chamber at 4°C. Primary antibodies consisted of chicken anti-NFH/-NFM/-NFL cocktail (1: 500; Aves Lab, Tigard, OR; NFH # AB2313552, NFM # AB2313554, and NFL # AB2313553), TUJ1 (1:500), SMI312 (1:500), rabbit anti-KHSRP (1:500; Novus Biologicals, Centennial, CO; #NBP1–18910), rabbit anti-REG3A (1:200; ThermoFisher; PA5–76091), chicken anti-GFP (1:500; Abcam), mouse anti-eIF2α (1:200; Cell Signaling Tech., Danvers, MA; 2103S), and rabbit anti-eIF2α^PS51^ (1:200; Cell Signaling Tech.; 9721S). After washes in 1x PBS, coverslips were incubated with a secondary antibody cocktail containing FITC-conjugated donkey anti-chicken, Cy3-conjugated donkey anti-mouse, or Cy5 conjugated donkey anti-rabbit diluted in block buffer (all at 1:500; Jackson ImmunoRes., West Grove, PA) for 1 h at room temperature. Coverslips were then washed 3 times in 1x PBS, rinsed with distilled H_2_O, and mounted with Prolong Gold.

For regeneration studies on mouse sciatic nerve and quantifying axonal content of KHSRP and REG3A in vivo, sciatic nerve segments were fixed, cryoprotected, cryosectioned, and stored as above. Tissue sections were brought to room temperature, equilibrated in 1x PBS and incubated with 20 mM Glycine for 30 min followed by 0.25 M NaBH_4_ for 30 min to quench autofluorescence. Sections were permeabilized in 0.3% Triton X-100 in 1x PBS for 15 min and blocked for 1 h at room temperature in 1x PBS containing 20 mM Glycine, 0.1% Triton X-100, and 10% Normal Donkey Serum. Primary antibodies consisted of, rabbit anti-KHSRP (1:200; Novus Biologicals, Centennial, CO; #NBP1–18910), rabbit anti-REG3A (1:200; ThermoFisher PA5–76091), rabbit anti-Stathmin-2/SCG10 (1:200; Novus Biologicals; #NBP1–49461), TUJ1 (1:200), SMI312 (1:200) (1:500 each), and chicken anti-GFP (Abcam, 1:200). The GFP antibody cross-reacts with BFP, so it was used to detect BFP signals from AAV-shRNA transduction. Samples were incubated overnight with primary antibodies in a humidified chamber at 4°C. The following day, samples were washed twice with 1X PBS and incubated with secondary antibodies consisted of FITC-conjugated donkey anti-chicken, Cy3-conjugated donkey anti-mouse, and Cy5-conjugated donkey anti-rabbit diluted in block buffer (all at 1:500; Jackson ImmunoRes.) for 1 h at room temperature. Sections were washed with three times with 1x PBS and once with H_2_O prior to mounting with Prolong Gold Antifade.

For neuromuscular junction (NMJ) analysis extensor digitorum longus (EDL) muscle was dissected and cleared of any connective tissue. Muscles were fixed for 20 min in 4% PFA, washed with PBS 3 times for 5 mins each and placed in 30% sucrose at 4°C. Muscles were lightly teased into smaller pieces and incubated with 1 μg/ml of Alexa488 conjugated α-Bungarotoxin overnight at 4°C with rocking (Thermo-Fisher, #B13422). Tissues were washed with PBS 3 times for 5 min each, then incubated with methanol at −20°C for 5 min and rinsed in PBS 3 times for 5 min each with rocking. Tissues were blocked for 1 h at room temperature with 2% BSA, 0.4% Triton X-100 in PBS then incubated overnight at 4°C with the with the following cocktail of primary antibodies diluted in blocking solution with rocking: rabbit anti-NF 200 (1:200; Millipore-Sigma, #N4142), mouse anti-synaptophysin (1:300; Millipore-Sigma, #MAB5258), and rabbit anti-synapsin-I (1:200; Millipore-Sigma, #AB1543P). The following day, tissues were rinsed 3 times 5 min each in PBS while rocking. Samples were then incubated in Cy3-conjugated donkey anti-rabbit and Cy3-conjugated donkey anti-mouse antibodies (1:500; Jackson ImmunoRes) overnight at 4°C with rocking. After rinsing in PBS, muscle fibers were spread into monolayers under a stereomicroscope and affixed to slides using Prolong Gold Antifade.

For DRG spot cultures, chamber wells were gently rinsed with PBS (37°C) and then fixed in 4% paraformaldehyde in PBS for 30 min at room temperature. After additional rinse in 1x PBS at room temperature, wells were incubated with primary and secondary antibodies as outlined above. Following this, chambers were removed and coverslips were mounted on glass slides using Prolong Gold.

All samples were mounted using Prolong Gold Antifade and samples were visualized using epifluorescent or confocal microscopy. Leica DMI6000 epifluorescent microscope with ORCA Flash ER CCD camera (Hamamatsu) was used for epifluorescent imaging. Confocal imaging for immunofluorescence was performed on a Leica SP8X microscope fitted with a galvanometer Z stage and HyD detectors; HC PL Apo 63x/1.4 NA objective (oil immersion) was used with acquisition parameters matched for individual experiments using *LASX* software. Z-stack images were post-processed by Leica Lightning Deconvolution integrated into *LASX* software. Deconvolved image stacks were projected into single plane images.

### Puromycinylation and proximity ligation analyses (puro-PLA)

For Puro-PLA, we used the Duolink PLA Multicolor Reagent Pack kit following the manufacturer’s instructions. DRG spot cultures were incubated with 2 μg/ml puromycin for 15 min at 37°C and fixed with 4% PFA in PBS. Neurons were permeabilized with 0.1% Triton X-100 and blocked with Duolink Blocking Solution. Neurons were incubated overnight at 4°C with red oligo conjugated primary antibodies (puromycin-1:500 and KHSRP- 1:500) and green oligo conjugated primary antibodies (puromycin- 1:500 and REG3A- 1:200) diluted in Duolink Antibody Diluent. The neurons were washed briefly with PBS and then incubated at 37°C for 1 h with Ligation Solution (a mixture between Duolink Ligation Buffer and Ligase). Neurons were washed three times with Duolink Wash Buffer A (0.15 M NaCl, 0.01 M Tris, 0.05% Tween 20). Neurons were incubated at 37°C for 100 min with Amplification Solution (a mixture between Duolink Amplification Buffer and Polymerase). Neurons were washed three times with Duolink Wash Buffer A. Neurons were incubated at 37°C for 30 min in Detection Buffer (diluted Duolink 5X Multicolor PLA Detection Buffer). Three washes with Duolink Wash Buffer B (0.1 M NaCl, 0.04 M Tris, 0.16 M Tris-HCl) were completed followed by a quick wash with 0.01x Duolink Wash Buffer B. Three washes with PBS were done. Neurons were post-fixed with 4% PFA and washed four times with PBS. Slides were blocked overnight at 4°C in 4% goat serum. Neurons were incubated at room temperature for 1 h in a cocktail of mouse Tuj1 (1:500) and mouse SMI312 (1:500) primary antibodies diluted in 4% goat serum. Three washes with PBS were completed. Neurons were incubated at room temperature for 30 min in DyLight 405 donkey anti-mouse (1:1,000) secondary antibody diluted in 4% goat serum. Three washes with PBS were completed. A quick wash with 0.01x Duolink Wash Buffer B was done and then neurons were stored in Duolink Wash Buffer B at 4°C until ready to be imaged. For the cycloheximide (CHX; Sigma) control groups, neurons were incubated with 150 μg/ml CHX at 37°C for 1 h before puromycin incubation. As an additional control, the Puro-PLA multiplexing protocol was completed omitting one of each of the oligo conjugated primary antibodies. Reactions were imaged without mounting on Leica Stellaris confocal microscope. There were no specific signals for the single antibody omitted reactions. 20–40 μM of neurofilament stained distal axons were analyzed using Fiji software. To acquire the number of puncta within the Puro-PLA fluorescent images, Intermodes Thresholding and the “Analyze Particles” function were utilized.

### Fluorescent recovery after photobleaching (FRAP)

FRAP was performed using a Leica confocal microscope fitted with an environmental chamber to maintain living cells at 37°C and 5% CO_2_. A 63x/NA 1.4 oil immersion objective was used for imaging with the pinhole set to 3 Airy units (AU) for pre-bleach, bleach, and post-bleach sequences to ensure entire thickness of axons was exposed to laser emission (*50*). Dissociated mouse DRG cultures transfected with GFP^MYR^5’/3’khsrp and/or mCherry^MYR^5’/3’reg3a, as indicated, were equilibrated in complete culture media that excluded phenol red. 72 h following transfection, GFP and/or mCherry expressing neurons were chosen for FRAP. A region of interest (ROI) in the most distal portion of the axon (40 x 40 μm, ≥ 250 μm from the soma) was photobleached with 488 nm (GFP) and/or 555 nm (mCherry) argon laser set at 100% power for 80 frames at 0.78 sec each. Pre-bleach and post-bleach signals were captured using 70% power for 488 and/or 555 nm laser line every 30 sec (2 images taken pre-bleach, and 30 images for post-bleach). Translation dependence of recovery was tested by pre-treating DRG cultures with 150 μg/ml CHX 15 min prior to photobleaching. For testing recREG3A- and Ca^2+^-dependent translation by FRAP, transfected DRG cultures were pretreated for 15 min with 5 μg/ml recREG3A, 3 μM BAPTA-AM, 3 μM BAPTA, or 90 μM GSK260614 PERK inhibitor. For combined treatments with recREG3A, DRG cultures were pretreated with inhibitors/chelators 15 min prior to recREG3A treatment.

### Protein fractionation and immunoblotting

DRG cultures were lysed, or sciatic nerves were minced and lysed in RIPA buffer (50 mM Tris-HCl [pH 8.0], 1% NP-40, 0.5% Sodium Deoxycholate, 0.1% SDS, 150 mM NaCl) plus protease inhibitors (Thermo Scientific; # A32965). Lysates were centrifuged at 20,000 x g for 15 min at 4°C. Protein concentrations were determined using Pierce BCA Protein Assay Kit (ThermoFisher; # 23227). After normalization for protein content, samples were denatured in Laemmli sample buffer [62.5 mM Tris (pH 6.8), 2% SDS, 10% glycerol, 1% β-mercaptoethanol, 0.01% bromophenol blue, 4 M Urea] at 95°C for 5 min followed by fractionation by standard SDS/PAGE. For separating soluble vs. insoluble cellular components, cultures were lysed and cleared by centrifugation as above. 2x Laemmli buffer + urea was added to supernatant to 1x and pellet was directly solubilized in 1x Laemmli buffer + urea; after denaturation at 95°C for 5 min, equivalent fractions of supernatant and pellet were fractionated by standard SDS/PAGE. Fractionated proteins were electrophoretically transferred to PVDF membranes (GE Healthcare Life Sciences, Marlborough, MA). Membranes were blocked in 5% non-fat dried milk powder (BioRad, Hercules, CA) in Tris-buffered saline (TBS) with 0.1% Tween-20 (TBST) for 1 h at room temperature. Blots were probed overnight at 4°C with the following antibodies diluted in TBST plus 3% BSA: rabbit anti-REG3A (Invitrogen; # PA5–76091), rabbit anti-ERK1 (Abcam; # ab109282), or mouse anti-HIS (Abcam; # ab18184). Membranes were washed in TBST and then incubated with horseradish peroxidase (HRP)-conjugated anti-rabbit IgG (1:2000; Cell Signaling Tech.) diluted in blocking buffer for 1 h at room temperature. Blots were washed in TBST and signals were detected by ECL Prime (GE Healthcare Life Sciences, Marlborough, MA).

### Behavioral analyses of axonal regeneration

Behavioral testing was conducted using adult male and female C57BL/6 J mice, including both *KHSRP*^−/−^ and *KHSRP^+/+^* genotypes that were age- and sex-matched. Mice underwent a unilateral sciatic nerve crush injury, with corresponding sham surgery to the contralateral side on d 0, and behavioral recordings were acquired at d 0 prior to sciatic nerve crush and every 3–4 d up to 28 d post-crush. A separate cohort of C57BL/6 J mice received a bilateral sciatic nerve injection of either shCtrl or shReg3a on d 0. On d 14 post-injection, these animals were subjected to a unilateral sciatic nerve crush, with a contralateral sham surgery. Behavioral recordings for this cohort were taken at d 0, prior to injection, and then every 3–4 d up to 21 d post-crush (35 d post-injection).

All mice were housed in standard clear plastic cages under controlled conditions (temperature 20–26°C, humidity 30–70%, lights on 07:00–19:00) throughout the duration of experiments and all recordings were done between 10:00 and 17:00 in the same room where the animals were housed using the BlackBox One instrument (BlackBox Bio, Cambridge, MA US). The animal containment chamber consisted of an 18 (l) × 18 (w) × 15 cm (h) black acrylic box that was closed on 4 sides and top; bottom of the box consisted of 5 mm thick borosilicate float glass for video recordings with 850 nm near-infrared (NIR) LED strips aligned perpendicular to 2 opposing edges of the glass and 2 separate 850-nm NIR LED strips positioned horizontally 10 cm below the glass floor. These NIR LEDs provide illumination of the animals for camera mounted beneath to recording paw placement/usage and animal movement as described (*18*).

Animals were placed into individual chambers within the device and allowed 10 min of habituation prior to each recording. After 5–10 min habituation period, animals were briefly removed to clean the glass surface of urine, feces, and any debris and then returned to individual chambers for a 20 min recording.

### Image analyses and processing

ImageJ was used to quantify protein and RNA levels in sciatic nerve tissues from optical planes of XYZ scans (*51*). Axon only signals were extracted via Colocalization plug-in that to project protein or RNA signals that overlap with axonal markers in each plane to a separate channel. These ‘axon only’ signals were then quantified in each XY plane of these axon only channels. Axon marker signal area was used to normalize signal intensities across the individual XY planes. The relative signal intensity was then averaged for all tiles in each biological replicate.

To assess regeneration in vivo, tile scans were post-processed by Straighten plugin for ImageJ (http://imagej.nih.gov/ij/). SCG10 fluorescence intensity was measured along the length of the nerve using ImageJ. Regeneration index was calculated by measuring the average SCG10 intensity in 500 μm bins across the length of the section starting at the crush site. The crush site was defined by the position along the nerve length with maximal SCG10 intensity (secondarily confirmed by DAPI signals and DIC images). Dissociated DRGs were immunostained with neurofilament antibodies as described above and axonal morphology was analyzed from epifluorescent tile scans using WIS-Neuromath to provide length and branch density information for each neuron (*52*).

For FRAP image sequences, raw images were analyzed for recovery in the bleached ROI using Leica Confocal Software. Percent fluorescent recovery was determined relative to pre-bleach and post-bleach signals, which were set at 100 and 0% to allow for comparisons between experiments and between neurons. For each treatment and construct tested, FRAP was analyzed for at least 7 neurons across 3 separate transfections.

### Statistical analyses

GraphPad Prism software package (La Jolla, CA) was used for statistical analyses. All experiments were performed in at least triplicate with statistical tests and post-hoc analyses as indicated in the results section. *P* ≤ 0.05 was considered as statistically significant.

R Studio (version 2026.01.2 + 418; Posit Software) was used to compare static Puro-PLA data and live-cell imaging values for KHSRP and REG3A vs. GFPMYR5’/3’Khsrp and mChMYR5’/3’Reg3a translation reporters, respectively. Per-growth cone signal intensities for KHSRP and REG3A were obtained from imaging-based measurements in both reporter (live-cell) and Puro-PLA (static) conditions. All analyses were performed on paired signal values for each growth cone. Initially, the Puro-PLA data were stratified into low (0–25%), intermediate (25–75%), and high (75–100%) KHSRP/REG3A ratio quartile groups based on the log-ratio distributions [log-ratio = log(KHSRP+ϵ) – log(REG3A + ϵ), where ϵ is a small constant (10−6) to avoid division by zero]. For visualization, KHSRP and REG3A Puro-PLA signals were normalized to the total signal per growth cone to obtain proportional values shown in Fig. 5F, non-normalized puncta data shown in fig. S6.

For analysis of signal distributions, we performed probability density estimation of the KHSRP and REG3A populations from the Puro-PLA and reporter datasets using the R density function for kernel density estimation (KDE) with default bandwidth selection. Density curves were compared qualitatively in fig. S6, B and C. An interaction proxy was defined as the product of KHSRP and REG3A signals (KHSRP × REG3A) for each growth cone of the PuroPLA and reporter studies. Density distributions of this proxy were computed in R using density and plotted as Fig. 5G. Differences between distributions of KHSRP, REG3A, and interaction proxy values between conditions were assessed in R using the Kolmogorov–Smirnov test.

For testing the signal relationship of KHSRP and REG3A between the Puro-PLA and reporter studies, KHSRP and REG3A signals were plotted as paired values in two-dimensional signal space using base R plotting functions (plot()). A locally weighted regression curve was fit to reporter (live-cell) data using the lowess function in R to define a trajectory representing coordinated signal coupling shown in Fig. 5H.

A threshold for trajectory adherence was defined based on the upper quantile (e.g., 95th percentile) of distances observed in reporter cells using R quantile function. Static cells exceeding this threshold were classified as off-trajectory and visualized in signal space using R plot function (fig. S6D). A reference trajectory was derived from reporter growth cones using lowess() applied to KHSRP vs. REG3A values. For each growth cone, the minimum Euclidean distance to the fitted trajectory was calculated by evaluating the distance between each point and the set of fitted curve coordinates. Distances to the trajectory were visualized as a histogram using R hist function (Fig. 6I) and compared between reporter and Puro-PLA growth cones using R boxplot function and the Wilcoxon rank-sum test in R (with exact = FALSE to account for ties) (fig. S6E).
