# Supplementary material for: An axon-intrinsic loop restricts nerve regeneration through axonal protein synthesis
Source: Sci Adv. 2026 Aug 26;12(35):eaed0049. doi: 10.1126/sciadv.aed0049 (PMC13510589; doi:10.1126/sciadv.aed0049)
Supplement: Supplementary file 1 [file sciadv.aed0049_sm.pdf]

Science Advances
AAAS

Supplementary Materials for

An axon-intrinsic loop restricts nerve regeneration through axonal protein synthesis

Courtney N. Buchanan *et al.*

Corresponding author: Jeffery L. Twiss, twiss@mailbox.sc.edu

Sci. Adv. **12**, eaed0049 (2026)
DOI: 10.1126/sciadv.aed0049

The PDF file includes:

Legends for movies S1 to S3
Figs. S1 to S6
Table S1

Other Supplementary Material for this manuscript includes the following:

Movies S1 to S3

MOVIES

Movie S1: ***Depletion of Reg3a accelerates axon regeneration.***
Representative video sequences for BFP signals in regenerating axons of shCntl-BFP- vs. shReg3a-BFP-transduced DRG spot cultures as indicated; scale bar = 10 µm.

Movie S2: ***Increased REG3A in injury conditioned DRG cultures is largely extracellular.***
Representative 3D renderings of REG3A protein and NF from dissociated L4-6 DRG cultures harvested from naïve vs. injury conditioned (‘Crush’) mice treated with heparinase as indicated; scale bar = 10 µm.

Movie S3: ***Increased REG3A in regenerating peripheral nerve is largely extracellular.***
Representative 3D renderings of REG3A protein and NF in naïve vs regenerating sciatic nerve (7 d post-crush); scale bar = 10 µm.

SUPPLEMENTARY FIGURES

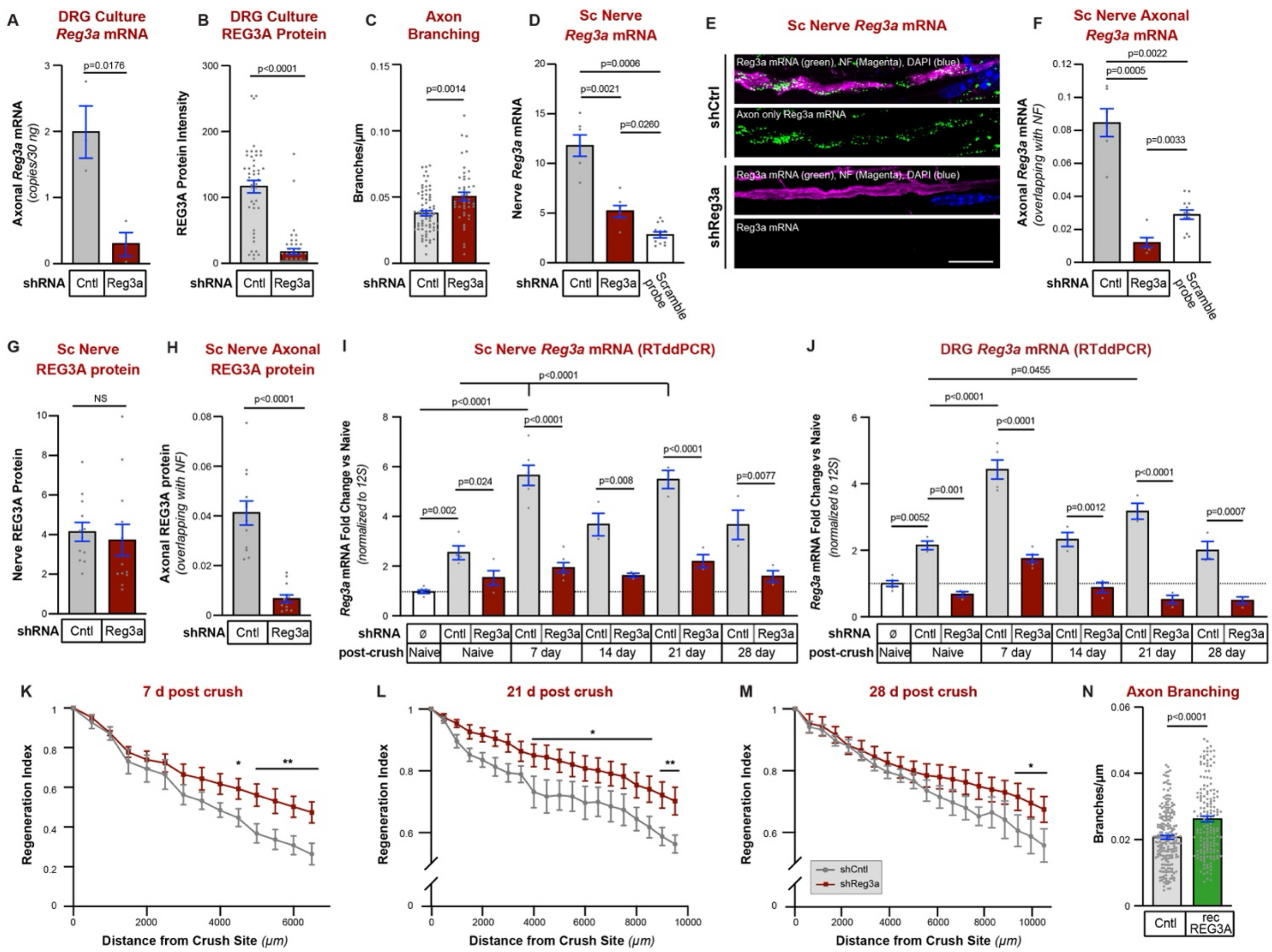

<u>Fig. S1</u>: *REG3A slows axonal growth.*

A-B, RTddPCR and immunofluorescence data show depletion of *Reg3a* mRNA (**A**) and REG3A protein (**B**) in shReg3a transduced DRG cultures as in Fig. 1C-D. Student's t-test, mean ± SEM (n = 3 mice for A and 45 neurons across ≥ 3 replicate cultures for B).

C, Quantitation of axon branching in AAV-shCntl vs. -shReg3a transduced DRG cultures from Fig. 1C-D shown as mean ± SEM. Student's t-test (n ≥ 75 neurons across ≥ 3 replicate cultures).

D-F, smFISH data for sciatic nerve sections from mice transduced with AAV-shRNAs show decreased overall *Reg3a* mRNA (**D**). Quantifying axon only *Reg3A* mRNA generated from subtracted images sequences to capture only the *Reg3a* signals overlapping with NF (see panel **E**) show that AAV-shReg3a specifically depletes the mRNA from axons (**F**); scramble smFISH probe was used as negative control). Student's t-test, mean ± SEM (n = 6 mice per group) scale bar = 10 µm.

G-H, Quantitation of REG3A immunoreactivity in nerve shows no difference between AAV-shReg3a and shCntl transduced mice for overall signal (**G**), but a significant reduction in the axonal REG3A with shReg3a transduction (**H**). Student's t-test, mean ± SEM (n = 6 mice per group).

I-J, RTddPCR for *Reg3a* mRNA in sciatic nerve (ScN) (**F**) and L4-6 DRG lysates (**G**) for naïve, non-transduced (ø) and shReg3a vs. shCntl transduced mice at 0-28 d post-ScN crush corresponding to Fig. 1E-J and Fig. 1H-J. One-way ANOVA with Tukey post-hoc analysis, mean ± SEM ($n \geq 3$ per group).
K-M, Regeneration indices based on SCG10 immunofluorescence for AAV-shRNA transduced mice at indicated post-crush intervals as in Fig. 1E-J. Two-way ANOVA with Sidak post-hoc analysis; mean ± SEM; * $p \leq 0.05$ ** $p \leq 0.005$ ($n \geq 5$ per group).
N, Quantitation of axon branching in recREG3A vs. Cntl treated DRG cultures from Fig. 1L shown as mean ± SEM. Student's t-test, mean ± SEM ($n \geq 163$ neurons across ≥ 3 replicate cultures).

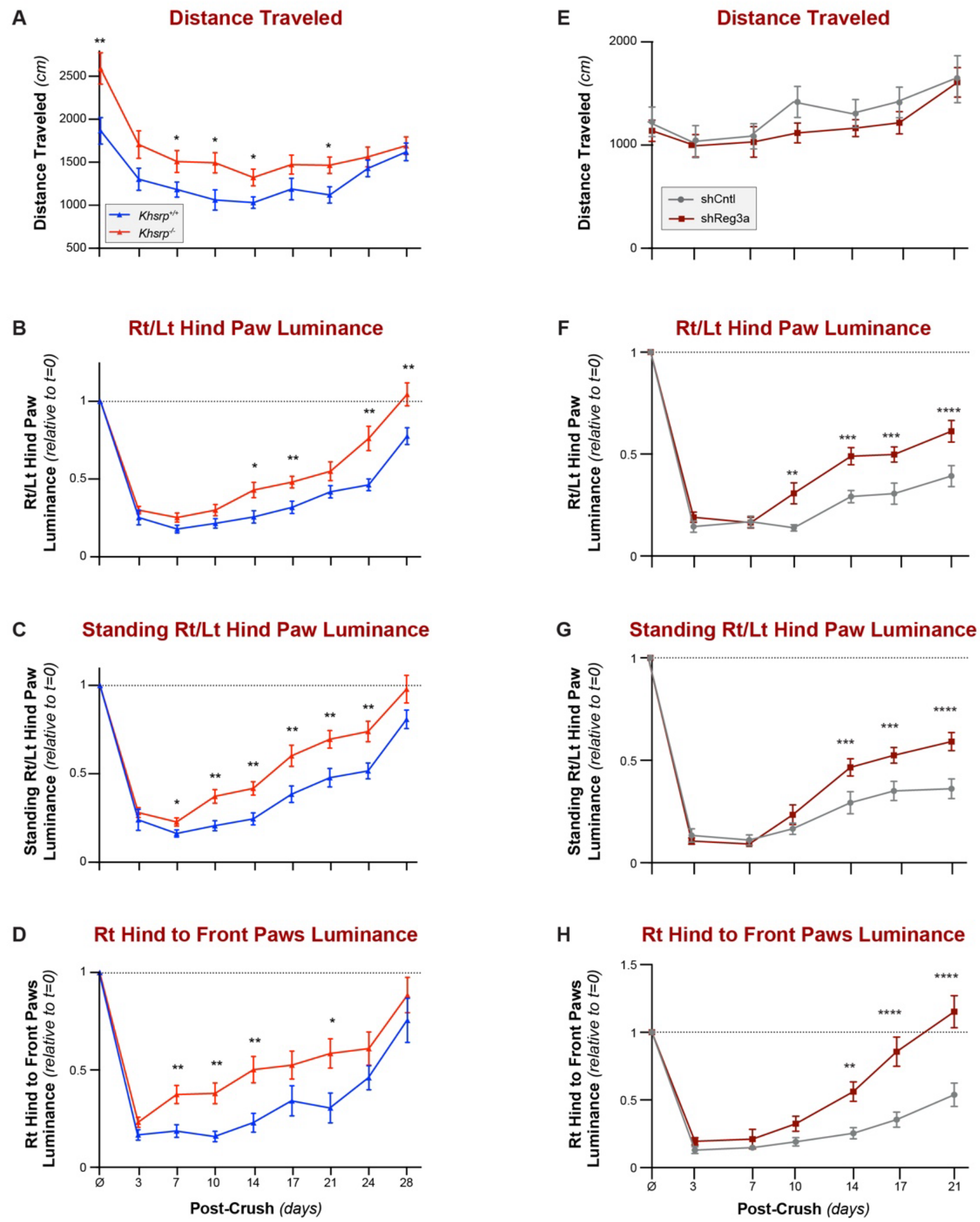

<u>Fig. S2</u>: *REG3A depletion accelerates functional recovery after nerve crush injury.*

A-D, BlackBox One analyses of *KHSRP*$^{-/-}$ compared to *KHSRP*$^{+/+}$ mice show increased distance traveled overall (**A**) and, after right-sided ScN crush, increased utilization of the hind paw ipsilateral to ScN crush injury compared to contralateral paw (**B-C**, Rt/Lt hind paw luminance and standing Rt/Lt hind paw luminance, respectively) and average front paws (**D**, Rt hind to front paws luminance). Two-way ANOVA with Tukey post-hoc analysis; mean ± SEM; * $p \leq 0.05$, ** $p \leq 0.005$ (n = 18 mice per genotype).

E-H, BlackBox One analyses for AAV-shCntl vs. -shReg3a transduced mice following unilateral (right) ScN crush injury. There is no difference in distance traveled (**E**), but *Reg3a* depleted mice show increased ipsilateral to injury vs. contralateral hind paws luminance (**F-G**, Rt/Lt hind paw and standing Rt/Lt hind paw luminance), ipsilateral to injury vs. average front paws luminance (**H**) compared to shCntl-transduced mice as in Fig. 1G. Two-way ANOVA with Tukey post-hoc analysis; mean ± SEM; * $p \leq 0.05$, ** $p \leq 0.005$, *** $p \leq 0.0005$, **** $p \leq 0.0001$ (n ≥ 10 mice per group).

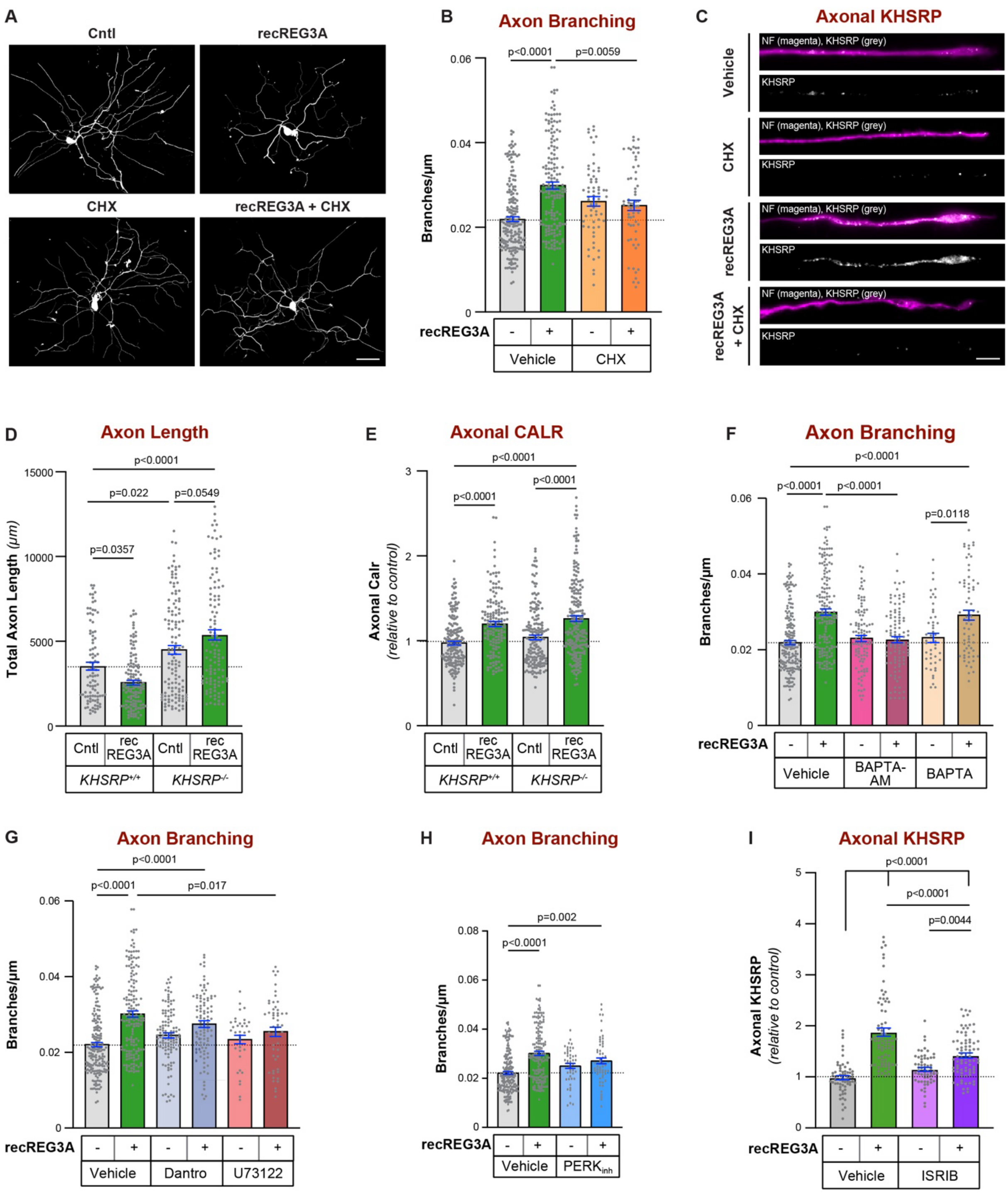

Fig. S3: ***REG3A regulates axon growth and KHSRP levels through modulation of axoplasmic Ca² levels.***

A-B, Representative images for NF + TuJ1 immunofluorescence (**A**) and axon branching quantitation (**B**) for DRGs treated with recREG3A ± cycloheximide (CHX) as in Fig. 2A. One-way ANOVA with Tukey post-hoc analysis, mean ± SEM (n ≥ 63 across ≥ 3 replicate cultures); scale bar = 100 µm.

C, Representative exposure matched images of terminal axons for DRG cultures treated as in A showing SMI312/TuJ1 and KHSRP immunofluorescence corresponding to Fig. 2B; scale bar = 5 µm.

D-E, Axon growth (**D**) and axonal Calreticulin protein (**E**) is shown for DRG neurons cultured from *KHSRP*$^{-/-}$ mice ± recREG3A treatment. Neurons lacking KHSRP do not show decrease in axon growth in response to recREG3A but still axonal Calreticulin levels increase in both *KHSRP*$^{+/+}$ and *KHSRP*$^{-/-}$ DRG neurons. One-way ANOVA with Tukey post-hoc analysis, mean ± SEM (n ≥ 94 neurons in D and n ≥ 143 in E across ≥ 3 replicate cultures).
F-G, Axon branching in DRG cultures ± recREG3A and BAPTA-AM vs. BAPTA (**F**), or dantrolene vs. U73122 (**G**) corresponding to Figure 3A, and E. One-way ANOVA with Tukey post-hoc analysis, mean ± SEM (n ≥ 46 neurons in F and ≥ 36 in G across ≥ 3 replicate cultures).
H, Axon branching in DRG cultures ± recREG3A PERK inhibitor corresponding to Figure 3G. One-way ANOVA with Tukey post-hoc analysis, mean ± SEM (n ≥ 60 neurons across ≥ 3 replicate cultures).
I, Axonal KHSRP levels for DRG neurons ± recREG3A in presence of ISRIB vs. vehicle. One-way ANOVA with Tukey post-hoc analysis, mean ± SEM (n ≥ 60).

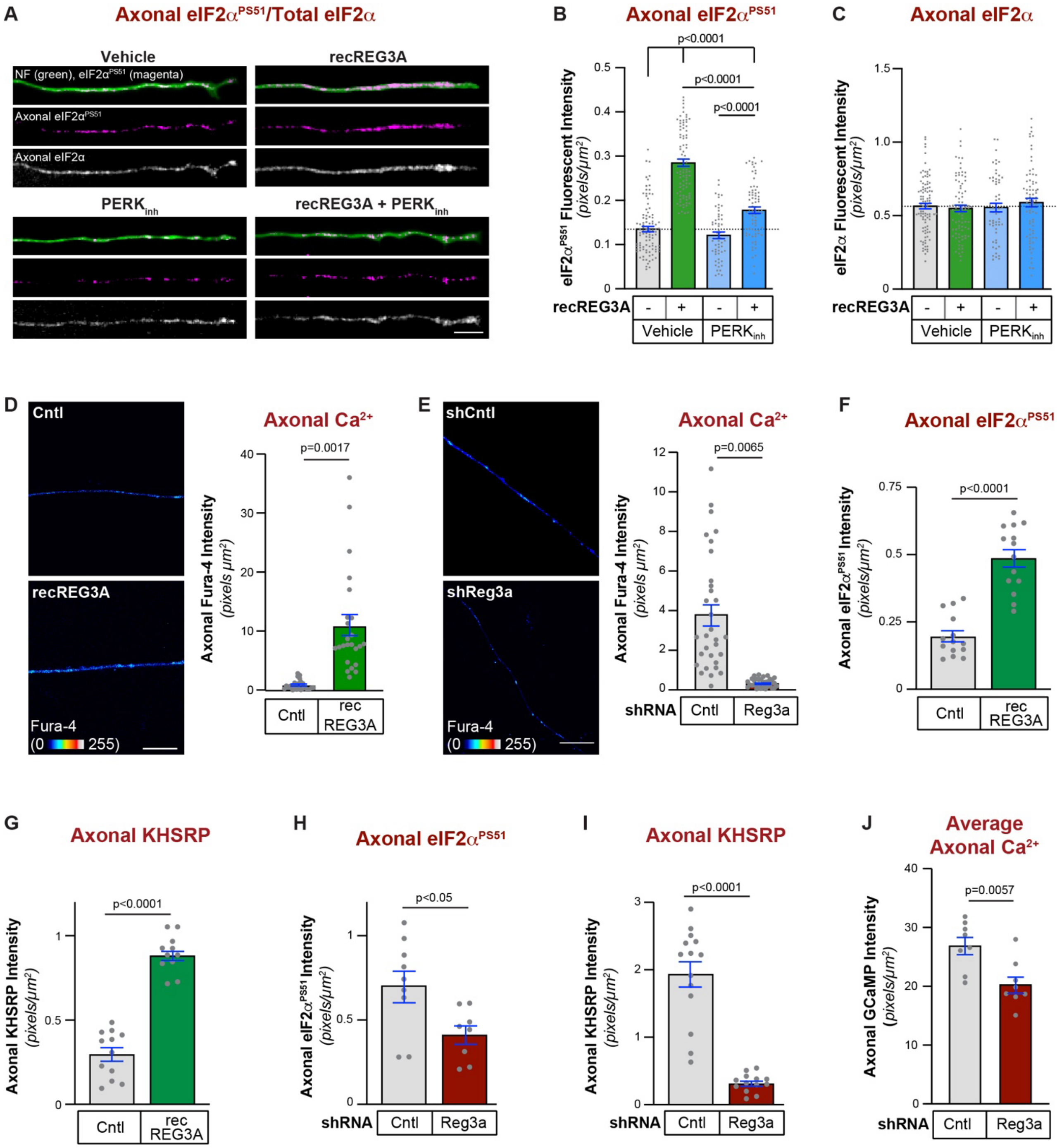

Fig. S4: ***REG3A regulates axon growth and KHSRP levels through modulation of axoplasmic Ca^{2} levels.*** **A-C,** Representative exposure-matched immunofluorescent images for eIFα and eIFα^{PS51} (**A**) and eIFα and eIFα^{PS51} levels in growth cones (**B-C**) of dissociated DRG cultures treated ± recREG3a with PERK inhibitor (PERK$_{INH}$) vs. vehicle (DMSO; n ≥ 60 neurons across ≥ 3 replicate cultures); scale bar = 5 µm. **D-E,** Representative images and quantitations for FURA-4 Ca^{2+}-indicator signals in axons of dissociated DRG cultures exposed to recREG3A (**D**) or transduced with AAV-shCntl vs. -shReg3a (**E**). Imaging

parameters for were set for recREG3A or shReg3A conditions with control parameters matched within each experiment. Student's t-test, mean ± SEM (n ≥ 24 neurons over ≥ 3 culture preparations); scale bar = 10 µm.
F-G, Quantitation of eIF2α^{PS51} (**F**) and KHSRP (**G**) immunofluorescence signals in distal axons of intact DRG spot cultures ± recREG3A vs. control for 1 h; mean ± SEM with P values by Student's t-test (n ≥ 75 neurons over ≥ 3 culture preparations).
H-I, Quantitation of eIF2α^{PS51} (**H**) and KHSRP (**I**) immunofluorescence signals in distal axons of AAV-shCntl vs. -shReg3a transduced DRG spot cultures at 16 h post transection; mean ± SEM with P values by Student's t-test (n ≥ 75 neurons over ≥ 3 culture preparations).
J, Average growth cone GCaMPs signal intensity regenerating axons of DRG spot cultures transduced with AAV-shCntl vs. -shReg3a shown as mean ± SEM; P value Student's t-test (n ≥ 75 neurons over ≥ 3 culture preparations).

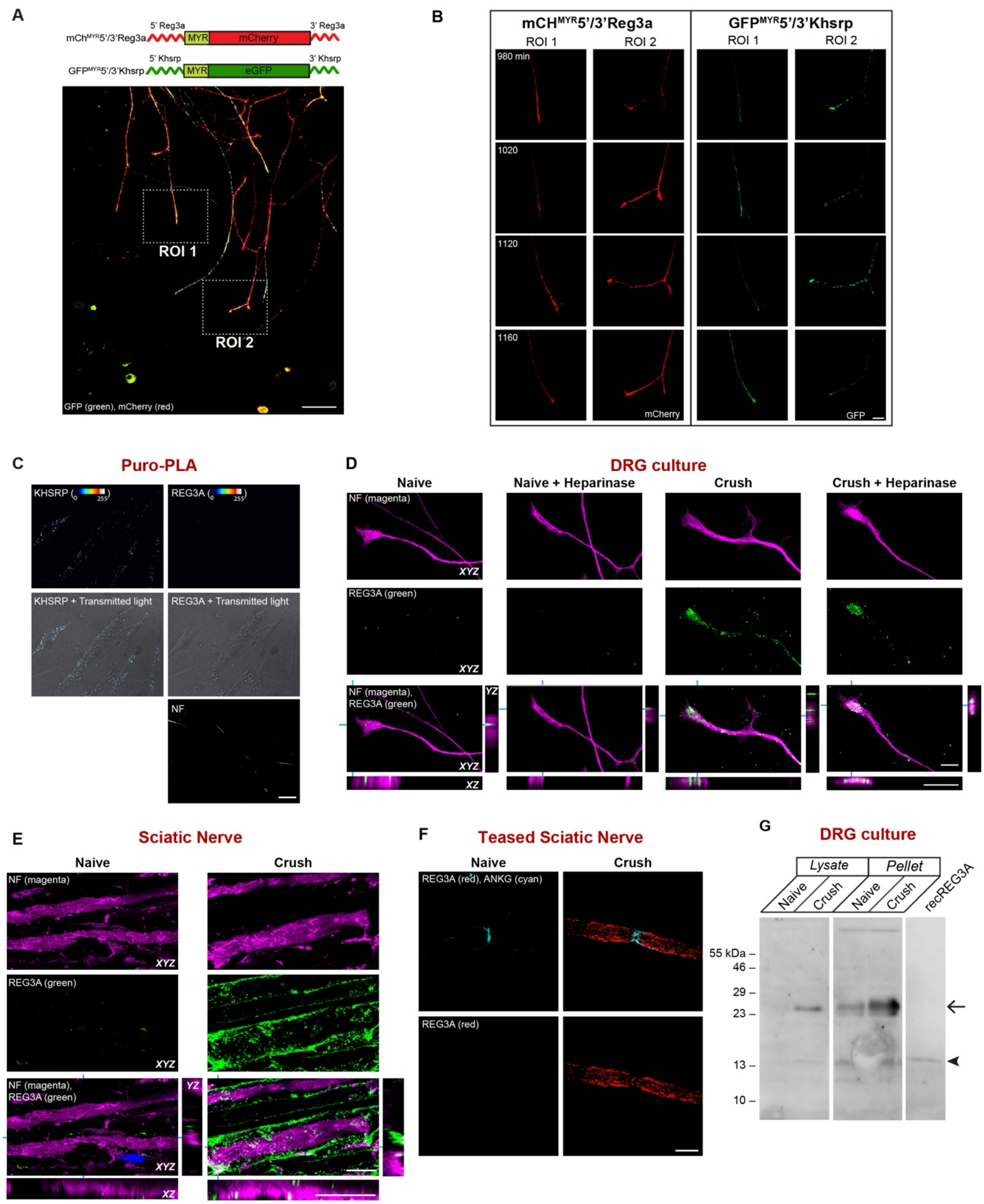

Fig. S5: ***Evidence for autocrine signaling by axonally-derived REG3A.***

A, Low magnification still image for regions of interest (ROI) from regenerating axons in DRG spot cultures transduced with AAV-GCaMP6s and co-transfected with GFP^{MYR}5'/3'Khsrp + mCh^{MYR}5'/3'Reg3a that were monitored for distal axon fluorescence in panel B; scale bar = 25 µm.

B, Representative images for GFP^{MYR}5'/3'Khsrp and mCh^{MYR}5'/3'Reg3a at indicated time points post-transection are shown for adjacent terminal axons indicated as ROI 1 and 2 in panel A; scale bar = 10 µm.
C, Representative images from DRG cultures used for Figure 5 D-F shows postive Puro-PLA signals for KHSRP in non-neuronal cells (likely Schwann cells) but only background for REG3A Puro-PLA signal (TL = transmitted light); scale bar = 20 µm.
D, Representative confocal XYZ maximum projection images for REG3A protein and NF from dissociated L4-6 DRG cultures harvested from naïve vs. 7 d injury-conditioned ('Crush') mice. These show increased REG3A protein in the injury-conditioned cultures. REG3A is largely concentrated extracellularly adjacent to the axonal membrane, as emphasized by the inset orthogonal XZ and YZ projections. Heparinase treatment decreases the membrane adjacent REG3A signal, particularly in the injury-conditioned DRG cultures. See Video S2 for 3D rendering of the XYZ images shown here; scale bars = 5 µm.
E, Representative confocal XYZ maximum projection images for optically isolated axons showing REG3A protein and NF in naïve vs. regenerating sciatic nerve (7 d post-crush) proximal to crush site with insets showing orthogonal XZ and YZ projections. Note that REG3A is increased in regenerating nerve sections and appears mostly outside of the axon as seen in panel D. See Video S3 for 3D rendering of the XYZ images shown here; scale bars = 10 µm.
F, Representative confocal XYZ projection of individual nerve fiber of teased nerve preparations from naïve vs. regenerating sciatic nerve (7 d post-crush) harvested proximal to crush site. There is a striking increase in REG3A in the crushed nerve preparation. Ankyrin G shows location of node of Ranvier (arrows) where prominent REG3A immunoreactivity is seen crossing the node; scale bar = 10 µm.
G, Representative immunoblot from naïve vs. injury-conditioned DRG cultures (7 d post-crush) confirms increase in REG3A signals after injury. REG3A is detected both in DRG lysates and insoluble debris ('pellet'), with much more insoluble than soluble REG3A protein (arrow). Note that recREG3A (arrowhead) runs at a lower molecular weight than the bulk of that isolated from DRGs, but there is a similar sized band in both the DRG lysates and pellet.

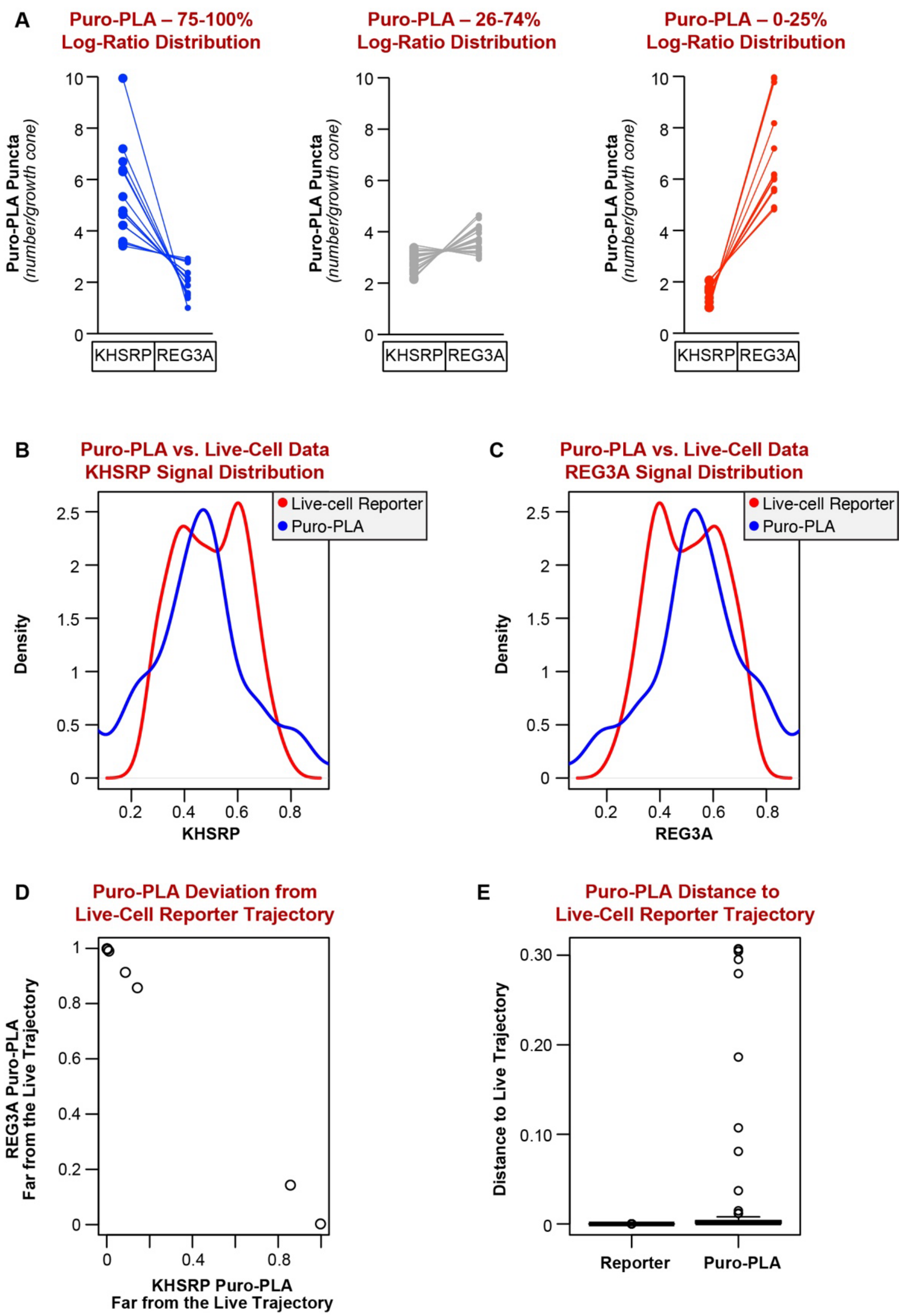

Fig. S6: ***Comparison of translation reporters and Puro-PLA for axonal REG3A and KHSRP.***

A, Puro-PLA punctae/growth cone for KHSRP and REG3A signals from individual growth cones based on quartiles from log-ratio distributions is shown for 75-100% (blue, left), 25-75% (grey, center), and 0-25% (red, right). These quartiles were used to generate proportion of total PLA signal graph shown in Figure 5F.

B-C, Density curves KHSRP (**B**) and REG3A (**C**) signals for individual growth cones in GFP^{MYR}5'/3'Khsrp–mCh^{MYR}5'/3'Reg3a reporters (red) and Puro-PLA (blue) conditions. Both conditions occupy a shared linear relationship, indicating a strong inverse coupling between the two signals.
D, Scatterplot of KHSRP versus REG3A for static cells classified as off-trajectory based on distance from the live-derived trajectory. These cells occupy regions outside the constrained linear relationship observed in Reporter cells, indicating altered coordination between signals. Approximately 84.4% of Puro-PLA growth cones fall within the defined GFP^{MYR}5'/3'Khsrp to mCh^{MYR}5'/3'Reg3a trajectory, defined as having a distance to the trajectory below a threshold of 0.02.
E, Distribution of distances from individual cells to the live-defined trajectory. Reporter cells exhibit minimal deviation, while Puro-PLA cells show a broader distribution with a subset of cells displaying substantially increased distances, indicating reduced coordination.

SUPPLEMENTARY TABLE S1: ***Summary of key reagents and sources.***

REAGENT or RESOURCE	SOURCE	IDENTIFIER
Antibodies		
Rabbit polyclonal anti-KHSRP antibody	Novus Biologicals	NBP1-18910
Rabbit polyclonal anti-REG3A antibody	Thermo Fisher	PA5-76091
Rabbit polyclonal anti-REG3A antibody	Thermo Scientific	PA5102577
Chicken anti-neurofilament (light)	Aves	Cat# NFL; RRID: AB_2313553
Chicken anti-neurofilament (medium)	Aves	Cat# NFM; RRID: AB_2313554
Chicken anti-neurofilament (heavy)	Aves	Cat# NFH; RRID: AB_2313552
Chicken polyclonal anti-GFP	Abcam	Ab13970
Mouse Monoclonal anti-eIF2a	Cell Signaling	2103S
Rabbit Anti-phosphorylated eIF2a (Ser51)	Cell Signaling	9721S
Mouse monoclonal anti-Tubulin beta-3 chain (TUJ1)	Biolegend	Cat# 801201
Mouse monoclonal anti-Neurofilament marker (SMI-312)	Biolegend	Cat# 837904
Rabbit polyclonal anti-Stathmin-2/STMN2 (SCG10)	Novus Biologicals	Cat# NBP1-49461
Mouse Anti-Puromycin	Kerafast	Cat# EQ0001
Anti-rabbit IgG, HRP-linked antibody	Cell Signaling	7074
Anti-mouse IgG, HRP-linked antibody	Cell Signaling	7076
Normal Donkey Serum	Jackson ImmunoResearch	017-000-121
Cy5-conjugated donkey anti-rabbit IgG (H+L)	Jackson ImmunoResearch	711-175-152
Cy3-conjugated donkey anti-mouse IgG (H+L)	Jackson ImmunoResearch	715-165-150
FITC-conjugated donkey anti-mouse IgG (H+L)	Jackson ImmunoResearch	715-095-150
FITC-conjugated donkey anti-chicken IgG (H+L)	Jackson ImmunoResearch	703-095-155
405-conjugated donkey anti-chicken IgG (H+L)	Jackson ImmunoResearch	703-475-155
DyLight 405 conjugated donkey anti-mouse	Jackson ImmunoResearch	715-475-151
Bacterial and virus strains		
pAAV9-hsynapsin1-axon-GCaMP6s-P2A-mRuby3	Addgene	RRID: 112005-AAV9 and 112005-AAV5
pAAV9-TagBFP2-U6-Ctrl shRNA	VectorBuilder	AAV9M(VB010000-0023jze)-C
pAAV9-TagBFP2-U6-Reg3a shRNA	VectorBuilder	AAV9M(VB221101-1415tar)-K1
**Biological samples**		
DRGs and Sciatic Nerve isolated from C57Bl/6 and *KHSRP*$^{-/-}$ mice	N/A	N/A
**Chemicals, peptides, and recombinant proteins**		
Recombinant Human REG3A protein (His Tag)	Sino Biological	Cat# 11235-H08H
Cycloheximide	Sigma	CAS# 66-81-9

BAPTA	Cayman Chemical	CAS# 85233-19-8
BAPTA-AM	Sigma	CAS# 126150-97-8
GSK2606414 (PERK inhibitor)	Tocris	CAS# 1337531-36-8
Dantrolene (RyR inhibitor)	Sigma	CAS# 14663-23-1
U73122 (PLC inhibitor)	Tocris	CAS# 112648-68-7
ISRIB (Integrated Stress Response Inhibitor)	Sigma	CAS# 1597403-47-8
Thapsigargin	Sigma	CAS# 67526-95-8
DMSO	Sigma	472301
SSC (20X), RNase-free	Thermo Fisher	AM9763
Dextran Sulfate	Sigma	S4030
E. Coli tRNA	Sigma	10109541001
Ribonucleoside Vanadyl Complex	NEB	S1402S
Bovine Serum Albumin	Sigma	10711454001
Formamide	Fisher	BP227
Triethylamine	Sigma	T58300
Acetic Anhydride	Fisher	A10
Salmon Sperm DNA	Sigma	D7656
Puromycin	Sigma	P8833
Heparinase I and III Blend from Flavobacterium heparinum	Sigma	H3917
Puromycin	Sigma	Cat# P8833

Critical commercial assays

RNeasy Microisolation kit	Qiagen	74034
Ribogreen RNA quantification	Fisher	R11490
Sensifast cDNA synthesis kit	Thomas Scientific	C755H66
QX200 ddPCR EvaGreen Supermix	BioRad	1864034
Automated droplet generation oil for EvaGreen	BioRad	1864112
BCA Protein Assay Kit	Thermo Fisher	23227
Duolink® PLA Multicolor Probemaker Kit- Red	Millipore-Sigma	DUO96010
Duolink® PLA Multicolor Probemaker Kit- Green	Millipore-Sigma	DUO96020
Duolink® PLA Multicolor Reagent Pack	Millipore-Sigma	DUO96000

Cell Culture Reagents

Poly-L-Lysine (50ug/mL)	Fisher	A005C
Poly-D-Lysine hydrobromide	Millipore-Sigma	P7280
Laminin (5ug/mL)	Millipore-Sigma	CC095
DMEM and Ham's F12 50/50, +L-Glutamine, + Phenol Red	Fisher	MT10090CV
DMEM and Ham's F12 50/50, +L-Glutamine, (- Phenol Red)	Fisher	MT16405CV
Fetal Bovine Serum	BioAbChem	72-0400
Cytosine β-D-Arabinofuranoside hydrochloride	Millipore-Sigma	C6645
Pen-Strep (Gibco 10,000 U/mL)	Fisher	15140122
L-glutamine	Fisher	A2916801
N21-Max Media Supplement	R&D Systems	AR008

Hibernate A	BrainBits	HA
Collagenase	Sigma	C-9697
Earle's Buffered Salt Solution, no phenol	Thermo Fisher	AAJ67559AE
Insulin (0.5mg/mL)	Sigma	I9278
Sodium Selenite (0.5 ug/mL)	Sigma	S5261
Transferrin (0.5 mg/mL)	Sigma	T8158
Putrescine (1.6 mg/mL)	Sigma	P5780
Progesterone (0.73 ug/mL)	Spectrum	P1834
Nucleofector kit for Primary Mammalian Neurons (SCN)	Lonza	VSPI-1003
Rat Neuron Nucleofector Kit	Lonza	VPG-1003
Prolong Gold Antifade with DAPI	Fisher	P36931
Prolong Gold Antifade without DAPI	Fisher	P36930

Experimental models: Organisms/strains

KHSRP knockout mice	(*49*)	N/A
C57BL/6J mice	Jackson Laboratories	RRID: IMSR_JAX:000664

Oligonucleotides

Stellaris probes against *Reg3a*	LGC Biosearch Tech	Genbank ID<br># NM_011259.1
Stellaris probes against Dapb - Quasar 570	LGC Biosearch Tech	SMF-1063-5<br>Target sequence:<br>TGATTCTTTAAAATATCCCG<br>ATAGGCAGTCGTAAATTGA
Stellaris probes against Dapb - Quasar 670	LGC Biosearch Tech	SMF-1065-5<br>Target sequence:<br>TGATTCTTTAAAATATCCCG<br>ATAGGCAGTCGTAAATTGA
Mitochondrial 12S RNA (*Mtrnr1*) PCR primer	Integrated DNA Technologies	sense –<br>GGCTACACCTTGACCTAACG<br>and antisense –<br>CCTTACCCCTTCTCGCTAATT<br>C
Reg3a PCR primer	Integrated DNA Technologies	sense –<br>TCTACAAGAGAGACAAGAT<br>GCTG and<br>antisense – AGCTGGTACGT<br>GGAGAGG

Recombinant DNA

GFP^{MYR}5'/3'khsrp	(*4*)	
mCh^{MYR}5'/3'reg3a	This manuscript	

Software and algorithms

FIJI/ImageJ	NIH	http://fiji.sc/cgi-bin/gitweb.cgi

NeuroMath	Weizmann Institute	http://www.weizmann.ac.il/vet/IC/software/wis-neuromath
Prism	GraphPad Software,	https://www.graphpad.com
Stellaris Probe Designer	BioSearch Technologies	https://oligos.biosearchtech.com/products/rna-fish/stellaris-probe-designer
SnapGene	SnapGene	https://www.snapgene.com
R Studio ver. 2026.01.2+418 (2026.01.2+418)	Posit Software	
